# Online GP-MPC Command Supervision for Robust Reinforcement Learning-Based Quadruped Locomotion

**DOI:** 10.3390/biomimetics11090641

**Published:** 2026-09-07

**Authors:** Seungyeon Lee, Hyunseok Yang

**Affiliations:** Department of Mechanical Engineering, Yonsei University, Seoul 03722, Republic of Korea; sehrgut010801@gmail.com

**Keywords:** quadruped robot, reinforcement learning, Gaussian Process, model predictive control, uneven terrain, command supervision

## Abstract

Reinforcement learning-based quadruped locomotion policies can exhibit command-tracking errors under terrain variations and unmodeled dynamics. This study proposes an online bounded Gaussian Process-enhanced model predictive control framework, termed Gaussian Process–Model Predictive Control–Reinforcement Learning(GP-MPC-RL), for command-level supervision of a pretrained locomotion policy. A frozen PPO policy generates the low-level locomotion behavior, while an acados-based MPC supervisor adjusts the velocity command using a nominal command-response model. An online Gaussian Process learns the one-step residual between the nominal prediction and measured robot response, and its uncertainty-weighted forward-velocity correction is incorporated into the MPC prediction. The framework was evaluated in Isaac Lab using a Unitree Go2 quadruped robot model over 20 paired rough-terrain trials at target velocities of 0.3, 0.5, and 0.7 m/s; GP-MPC-RL reduced the mean forward-velocity root mean square error (RMSE) relative to PPO by 38.3%, 21.3%, and 10.1%, respectively. Under a 5 kg payload, GP-MPC-RL reduced velocity RMSE by 29.0% relative to MPC-RL and reduced the 0–5 kg payload-induced degradation by 53.0% (*p* = 0.019). The average supervisor computation time was 0.112 ms. These results indicate that GP residual adaptation is particularly effective when the nominal command-response model becomes inaccurate, improving robustness without retraining the underlying locomotion policy.

## 1. Introduction

Quadruped robots have attracted considerable attention in applications such as inspection, exploration, search and rescue, and industrial automation because they can traverse environments that are difficult for conventional wheeled platforms, including rough terrain, steep slopes, irregular steps, and discontinuous ground surfaces [1,2,3,4]. Maintaining stable locomotion in these environments remains challenging because terrain geometry, friction, and contact conditions continuously change during locomotion. Mountain goats are well known for their ability to move across steep and rocky terrain by continuously adjusting body posture and limb loading according to the interaction between the feet and the ground rather than relying on predefined movement patterns [5,6]. In this study, this adaptive behavior is considered as a biological motivation for continuously adjusting locomotion commands in response to environmental changes rather than directly reproducing biological locomotion. Accordingly, the objective of the Gaussian Process (GP) component is not to replace a well-performing nominal Model Predictive Control (MPC) at every operating point but to provide adaptive residual compensation when the nominal command-response model becomes inaccurate. This distinction is particularly relevant when the robot experiences terrain- or payload-induced dynamics that differ from those represented by the nominal model.

Recent advances in reinforcement learning (RL) have enabled quadruped robots to perform agile locomotion over a wide range of terrains. Policies trained in simulation can generate coordinated whole-body motions, recover from external disturbances, and operate in challenging natural environments without manually designed gait transitions [7,8,9,10]. In particular, Proximal Policy Optimization (PPO) has become one of the most widely adopted RL algorithms for quadruped locomotion because of its stable learning performance in continuous control tasks [11]. Nevertheless, most RL policies are optimized using the environments encountered during training. Their performance may therefore vary when the robot experiences terrain conditions with different friction properties, contact characteristics, or surface geometries. Although domain randomization and adaptation techniques have been proposed to alleviate this problem, exposing the policy to every possible terrain during training remains difficult [12,13]. MPC has been widely adopted to predict future system behavior and determine control inputs over a finite prediction horizon [14,15]. The prediction performance of MPC, however, depends on the accuracy of the prediction model. For an RL-controlled quadruped, the closed-loop response is influenced by the learned policy, actuator dynamics, contact interactions, and terrain conditions. Consequently, a nominal prediction model identified under limited operating conditions may not accurately represent the robot’s behavior on uneven terrain. GP regression provides a data-driven approach for learning the discrepancy between nominal model predictions and measured system responses [16,17]. In this work, an online bounded GP is used to update the residual model from newly observed transitions while limiting the size of the retained dataset. GP regression was selected because it can represent nonlinear residual behavior from a compact dataset while also providing predictive uncertainty, which is used to attenuate unreliable residual corrections. The resulting correction can be incorporated into the supervisory prediction without modifying the low-level locomotion policy. This provides an opportunity to investigate whether command-level prediction can better represent terrain-dependent robot behavior while preserving the original RL controller.

Previous studies have extensively investigated RL-based locomotion, MPC-based locomotion control, and GP-based residual learning. However, comparatively few studies have isolated the contribution of online GP residual adaptation within a command-level MPC supervisor while keeping the underlying RL locomotion policy fixed. This separation is important because it allows the effect of nominal MPC supervision to be distinguished from the additional benefit provided by online residual learning. The central hypothesis of this study is that online residual adaptation becomes most beneficial when the measured closed-loop response deviates from the nominal command-response prediction while providing limited additional benefit when the nominal model already represents the operating condition sufficiently well. Furthermore, the contribution of GP residual learning has rarely been evaluated independently while maintaining an identical low-level locomotion policy. Unlike residual-control approaches that directly modify low-level control actions, the proposed method uses GP residual adaptation only within the prediction model of an upper-level command supervisor while keeping the pretrained PPO policy unchanged.

Therefore, this paper proposes a GP-MPC command-supervision framework for reinforcement learning-based quadruped locomotion on uneven terrain. A frozen PPO policy generates the low-level locomotion actions, while an upper-level GP-MPC supervisor modifies the velocity command using a GP-corrected prediction model. A nominal command-response model describes the dominant closed-loop behavior, while an online bounded multi-output GP is updated from the residual between the nominal one-step prediction and the measured robot response. The GP prediction is used to construct an uncertainty-weighted residual correction, which is incorporated into the MPC prediction before the optimized command is supplied to the frozen PPO policy. Throughout all comparative experiments, the same pretrained PPO policy is maintained so that the contributions of command-level MPC supervision and GP residual adaptation can be evaluated separately. The framework therefore adapts the supervisory prediction to model mismatch without retraining or directly modifying the underlying locomotion policy.

The main contributions of this work are summarized as follows.

1. A hierarchical command-supervision framework is proposed in which a pretrained and frozen PPO locomotion policy is retained unchanged while an upper-level MPC adjusts the velocity command without modifying the low-level policy.
2. An online bounded multi-output GP residual model is incorporated into the supervisory prediction to compensate for discrepancies between the nominal command-response model and the measured robot response, with predictive uncertainty used to attenuate unreliable residual corrections.
3. The contributions of nominal MPC supervision and online GP residual adaptation are isolated through paired comparisons among PPO, MPC-RL, and GP-MPC-RL while maintaining an identical pretrained locomotion policy and experimental conditions.
4. The framework is evaluated over 20 paired rough-terrain trials per operating condition at target velocities of 0.3, 0.5, and 0.7 m/s and under 0, 2, and 5 kg payload conditions, enabling evaluation of both nominal tracking performance and robustness to increased model mismatch.

The remainder of this paper is organized as follows. Section 2 presents the overall control framework, nominal command-response model, online bounded GP residual model, and GP-MPC supervisor. Section 3 describes the simulation environment, controller configuration, experimental scenarios, and evaluation metrics. Section 4 presents a separate terrain-conditioned GP-MPC implementation as an additional validation study. Section 5 reports the experimental results and discusses the observed performance and limitations. Finally, Section 6 summarizes the conclusions and future work.

## 2. Methodology

This section describes the proposed GP-MPC command supervision framework for reinforcement learning-based quadruped locomotion on uneven terrain. The framework is organized into two hierarchical control layers: a high-level command supervisor and a low-level locomotion controller. The high-level controller predicts the future robot response, updates an online bounded GP residual model using measured transition errors, and optimizes the commanded velocity through MPC. The optimized velocity command is executed by a frozen reinforcement learning policy, inverse kinematics (IK), and a proportional–derivative (PD) controller [18,19]. The following subsections describe each component of the proposed framework in detail.

### 2.1. Overall GP-MPC Framework

**Figure 1:**
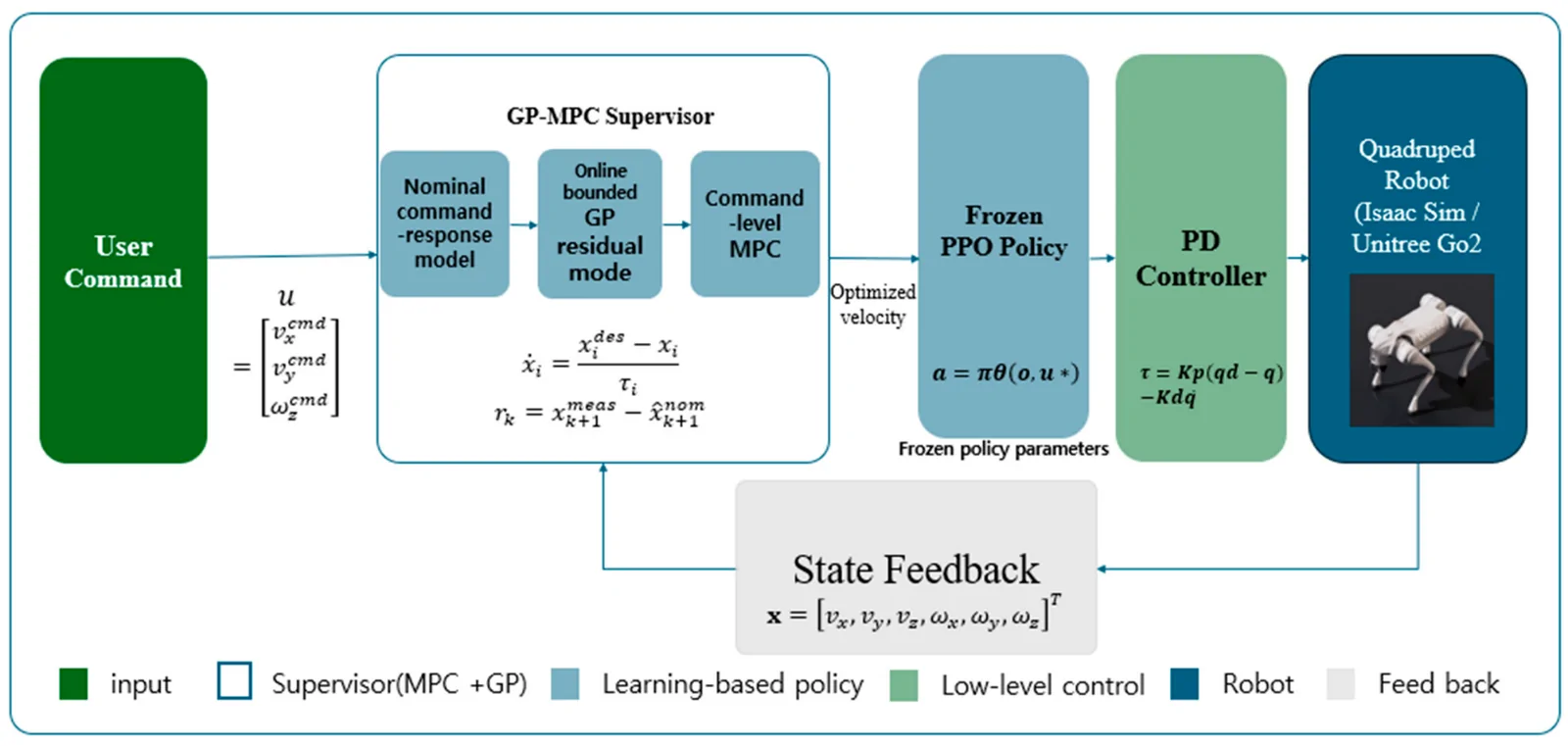
Overall architecture of the proposed online bounded GP-MPC command supervision framework. Arrows indicate the forward command/data flow and the feedback path used by the supervisor. The asterisk (*) in u* denotes the optimized velocity command generated by the GP-MPC supervisor and supplied to the frozen PPO policy.

Figure 1 illustrates the overall architecture of the proposed GP-MPC framework. Unlike conventional learning-based locomotion methods that directly modify the policy network, the proposed approach preserves the trained reinforcement learning policy and optimizes only the high-level velocity command. This hierarchical design separates command generation from low-level motion execution, allowing the learned locomotion controller to remain unchanged while enabling model-based adaptation of the supervisory command.

The framework consists of three sequential modules within the GP-MPC supervisor. First, a nominal command-response model predicts the future robot state from the current state and velocity command. Second, an online bounded multi-output GP residual model estimates the one-step prediction error associated with terrain-dependent contact conditions, payload-induced dynamics variations, and other unmodeled effects and is updated using newly observed transitions. Finally, a command-level MPC computes the optimal velocity command using the GP-corrected prediction model. Forward, lateral, and yaw-rate commands, with the lateral reference fixed to zero, were used in the present experiments.

The user specifies the desired forward velocity and yaw rate,

(1) $$u_{k}^{ref}=\left[\begin{array}{c} v_{x,k}^{ref}\\ v_{y,k}^{ref}\\ \omega_{z,k}^{ref} \end{array}\right],$$

where $v_{x}^{cmd}$ and $\omega_{z}^{cmd}$ denote the desired forward velocity and yaw rate, respectively.

The optimized command generated by the GP-MPC supervisor is denoted as

(2) $$u_{k}^{*}=\left[\begin{array}{c} v_{x,k}^{*}\\ v_{y,k}^{*}\\ \omega_{z,k}^{*} \end{array}\right],$$

The optimized command $u_{k}^{*}$ replaces the corresponding user-command input supplied to the frozen PPO policy. The supervisor therefore modifies only the high-level velocity command, while the PPO parameters and the underlying locomotion policy remain unchanged. During locomotion, the measured robot state is continuously fed back to the supervisor for state prediction, residual computation, online GP updating, and command optimization.

### 2.2. Low-Level Kinematics and Joint Control

**Figure 2:**
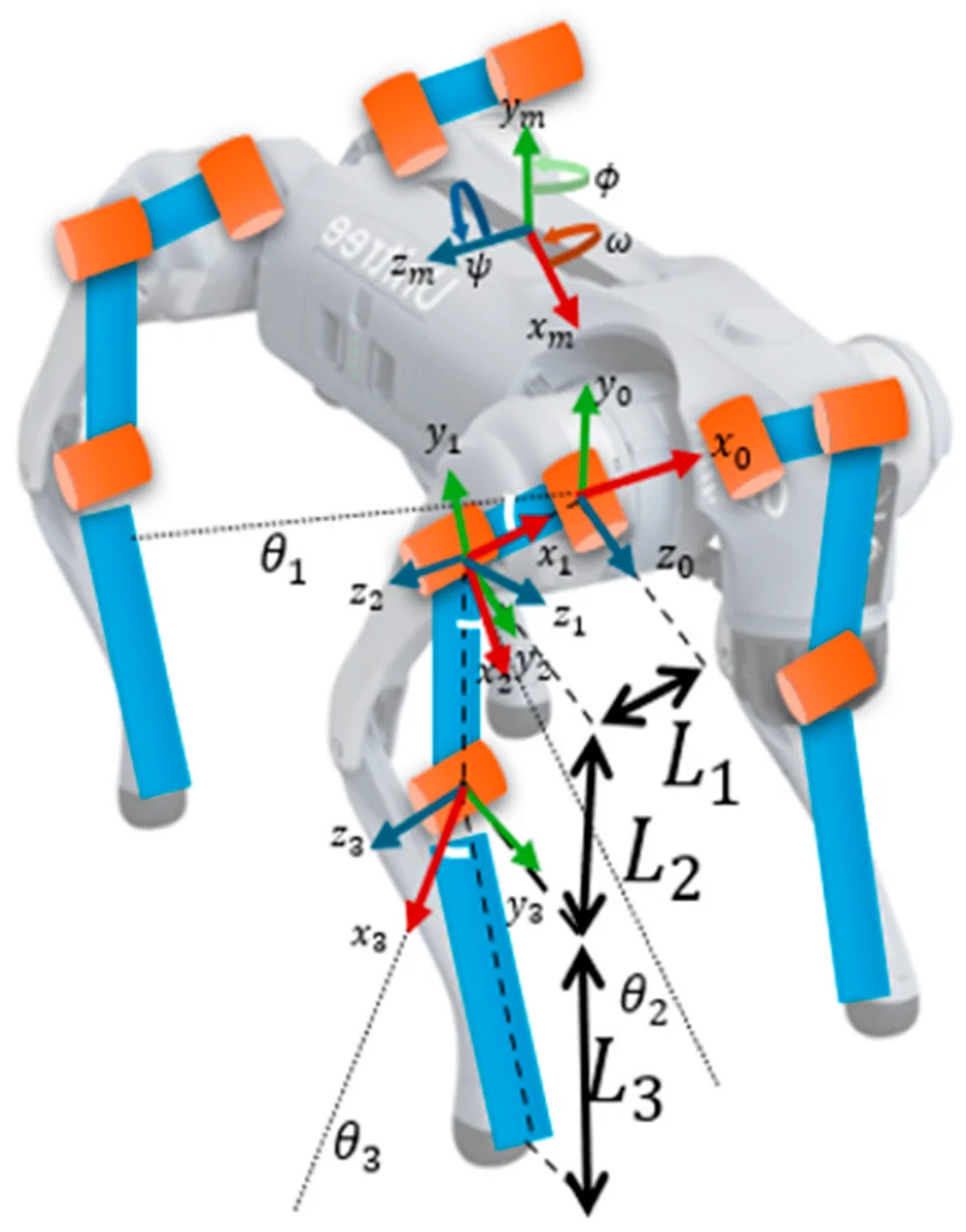
Kinematic model and low-level joint control structure of a representative quadruped leg. Each leg is modeled as a serial mechanism with three degrees of freedom. L1–L3 denote representative link lengths, θ1–θ3 denote joint angles, and the colored axes indicate local coordinate frames.

The optimized velocity command generated by the supervisory controller is supplied to the frozen PPO locomotion policy. The policy then generates the low-level locomotion actions, which are executed through the IK and PD control layers. The same PPO policy, IK formulation, and PD gains are used for PPO, MPC-RL, and GP-MPC-RL so that the compared methods differ only in their command-level supervisory strategy. Figure 2 illustrates the kinematic model adopted for one representative leg of the quadruped robot. Since all four legs share the same kinematic structure, only one leg is shown for clarity.

Each leg is modeled as a serial kinematic chain with three degrees of freedom composed of the hip-abduction/adduction, hip-flexion/extension, and knee-flexion/extension joints. A body-fixed coordinate frame is attached to the robot torso, while local coordinate frames are assigned to the individual joints [20]. The corresponding link lengths are denoted by L1, L2, and L3.

Given the desired foot position $p_{f}={\left[x_{f},y_{f},z_{f}\right]}^{T}$, the corresponding hip-abduction/adduction, hip-flexion/extension, and knee-flexion/extension joint angles are obtained using the following closed-form inverse-kinematics relations:

(3) $$\theta_{1}=-atan2\left(-y_{4},x_{4}\right)-atan2\left(\sqrt{x_{4}^{2}+y_{4}^{2}-L_{1}^{2}}-L_{1}\right)$$

(4) $$\theta_{2}=-atan2\left(z_{4},\sqrt{x_{4}^{2}+y_{4}^{2}-L_{1}^{2}}\right)-atan2\left(L_{3}sin\left(\theta_{3}\right),L_{2}+L_{3}cos\left(\theta_{3}\right)\right)$$

(5) $$\theta_{2}=-atan2\left(\sqrt{1-D^{2}},D\right)\left(legs\;for\;2\;and\;4\right)$$

(6) $$\theta_{3}=-atan2\left(-\sqrt{1-D^{2}},D\right)\left(legs\;for\;1\;and\;3\right)$$

(7) $$D=(x_{4}^{2}+y_{4}^{2}-L_{1}^{2}+z_{4}^{2}-L_{2}^{2}-L_{3}^{2})/(2L_{2}L_{3})$$

where $\theta_{1}$, $\theta_{2}$, and $\theta_{3}$ denote the hip-abduction/adduction, hip-flexion/extension, and knee-flexion/extension joint angles, respectively. The sign convention in (5) and (6) accounts for the mirrored kinematic configurations of the left and right legs.

(8) TFB=THB ∗T1Hq1∗T21q2 ∗T32q3,

where THB, T1Hq1, T21q2, and T32q3 denote the successive homogeneous transformations along the leg kinematic chain. The resulting transformation TFB describes the foot pose with respect to the robot body frame.

(9) $$p_{F}=f\left(q\right),$$

where $f\left(\cdot \right)$ denotes the nonlinear forward kinematic mapping.

Given the desired foothold generated by the locomotion controller, the desired joint configuration is computed through inverse kinematics,

(10) $$q^{des}=f^{-1}\left(p^{des}\right),$$

where $p^{des}$ represents the desired foot position.

To improve numerical stability, the joint update is calculated using the Jacobian pseudo-inverse,

(11) $$\Delta q=J^{†}\left(p^{des}-p\right),$$

where $J^{†}$ denotes the Moore–Penrose pseudo-inverse of the Jacobian matrix.

Finally, the desired joint positions are tracked using a PD controller,

(12) $$\tau =K_{p}\left(q^{des}-q\right)-K_{d}\dot{q},$$

where q, $\dot{q}$, and τ denote the measured joint positions, joint velocities, and commanded joint torques, respectively.

The PPO policy, inverse-kinematics solver, and PD controller are identical for all compared methods. Therefore, differences in locomotion performance are attributed solely to the command-level supervisory strategy.

### 2.3. Nominal Command-Response Model

The proposed GP-MPC framework employs a nominal command-response model to predict the short-term closed-loop response of the quadruped robot. Rather than modeling the complete rigid-body and contact dynamics, the nominal model approximates the relationship between the supervisory velocity command and the measured robot response. This simplification reduces the computational cost of online prediction while preserving the dominant command-response behavior required by the high-level supervisor. The discrepancy between this nominal prediction and the measured response is subsequently learned by the online GP residual model.

**Figure 3:**
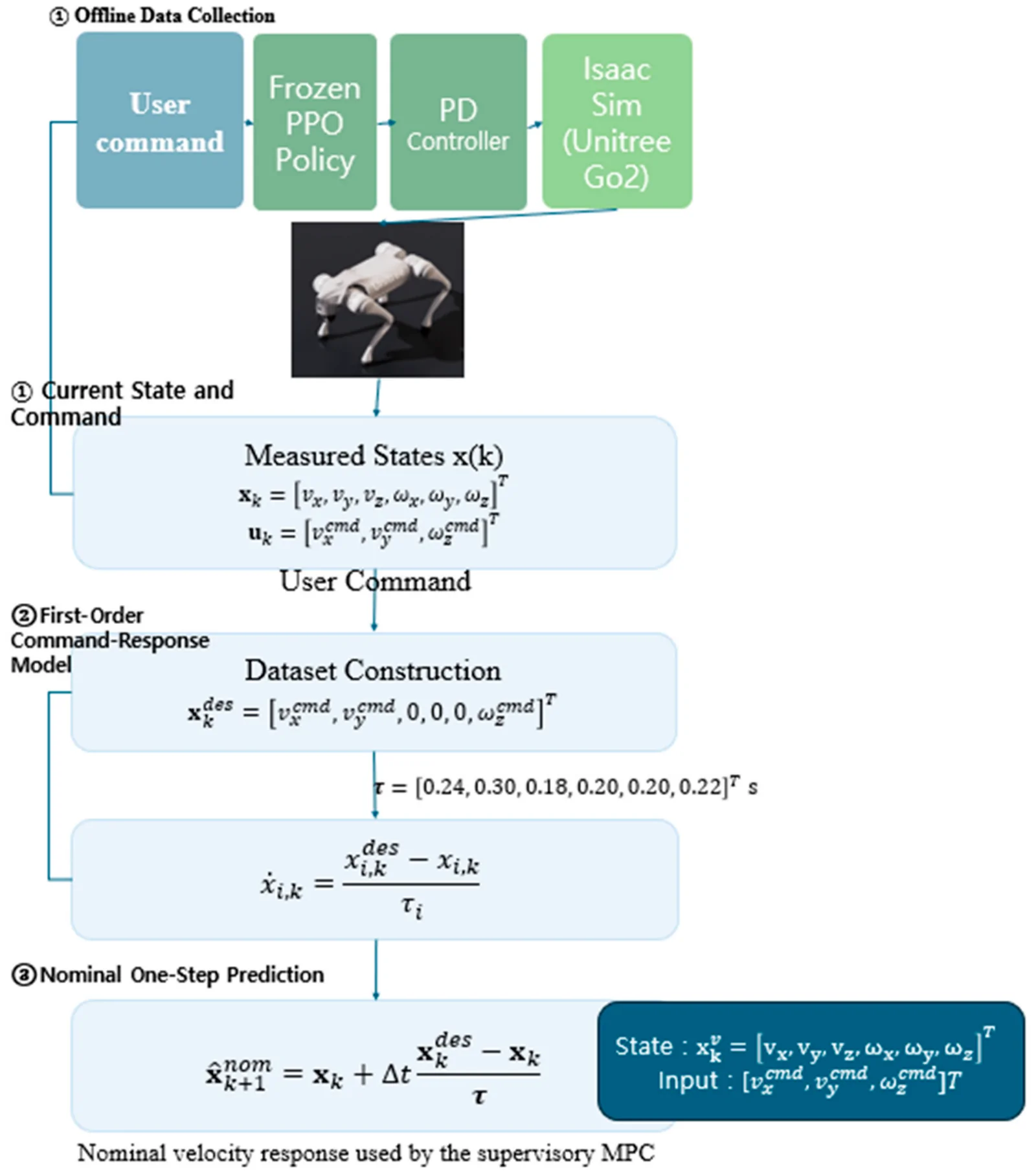
Nominal command-response model used for supervisory prediction.

The nominal command-response model approximates the short-term closed-loop velocity response of the frozen PPO locomotion controller. Rather than identifying a full rigid-body model, the supervisor uses a six-dimensional first-order response model, The nominal command-response prediction process is summarized in Figure 3.

(13) $$x_{k}={\left[v_{x,k},v_{y,k},v_{z,k},\omega_{x,k},\omega_{y,k},\omega_{z,k}\right]}^{T},$$

where $v_{x}$, $v_{y}$, and $v_{z}$ denote the body linear velocities, and $\omega_{x}$, $\omega_{y}$, and $\omega_{z}$ denote the body angular velocities. The supervisory command is defined as

(14) $$u_{k}={\left[v_{x,k}^{cmd},v_{y,k}^{cmd}\;,\omega_{z,k}^{cmd}\right]}^{T},$$

and the corresponding desired velocity state is

(15) $$x_{k}^{des}={\left[v_{x,k}^{cmd},v_{y,k}^{cmd},\;0,0,0,\omega_{z,k}^{cmd}\right]}^{T}.$$

The nominal closed-loop response is modeled independently for each velocity component as

(16) $${\dot{x}}_{i,k}=\frac{x_{i,k}^{des}-x_{i,k}}{\tau_{i}},$$

with

(17) $$\tau ={\left[0.24,\;0.30,\;0.18,\;0.20,\;0.20,\;0.22\right]}^{T}\;s.$$

Using the supervisory sampling interval Δt, the nominal one-step prediction is

(18) $${\hat{x}}_{k+1}^{nom}=x_{k}+\Delta t\left[\frac{x_{k}^{des}-x_{k}}{\tau }\right].$$

where the division is performed element-wise. This compact model captures the dominant command-response behavior required by the supervisory MPC while avoiding the computational cost of modeling the full rigid-body and contact dynamics. The remaining discrepancy between the nominal one-step prediction and the measured velocity response is learned online by the bounded GP residual model described in Section 2.4.

### 2.4. GP Residual

**Figure 4:**
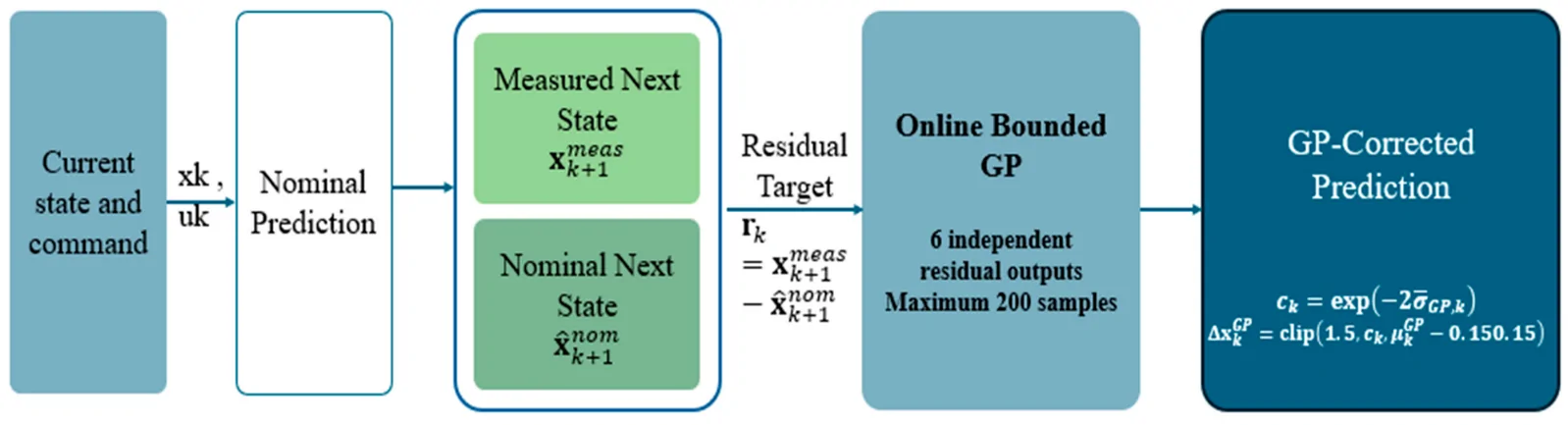
Online bounded GP residual learning for prediction correction.

Although the nominal command-response model captures the dominant closed-loop dynamics, prediction errors remain due to terrain-dependent contact conditions, actuator dynamics, payload-induced dynamics and other unmodeled effects. To compensate for these errors, a GP residual model is employed to learn the discrepancy between the nominal prediction and the measured robot response. The online residual-learning and prediction-correction process is illustrated in Figure 4.

At each supervisory update, the GP input is constructed as

(19) $$z_{k}^{GP}={\left[\begin{array}{ccc} s_{k}^{T} & u_{k}^{T} & \eta_{k}^{T} \end{array}\right]}^{T}\in R^{19},$$

where $s_{k}\in R^{12}$ denotes the robot-state input available to the supervisor, $u_{k}\in R^{3}$ is the supervisory velocity command, and $\eta_{k}\in R^{4}$ denotes the terrain/context input. For the main PPO, MPC-RL, and GP-MPC-RL comparisons in Section 5, the terrain/context input is set to zero so that the online GP correction is learned directly from the observed command-response mismatch. The terrain-conditioned formulation presented in Section 4 is treated separately. The nominal one-step velocity prediction is obtained from the command-response model defined in Section 2.3. After the next measured velocity state becomes available, the six-dimensional residual is calculated as

(20) $$r_{k}=x_{k+1}^{meas}-{\hat{x}}_{k+1}^{nom},r_{k}\in R^{6},$$

where

(21) $$r_{k}={\left[r_{vx,k},r_{vy,k},r_{vz,k},r_{\omega x,k},r_{\omega y,k},r_{\omega z,k}\right]}^{T}.$$

To suppress unusually large transition errors before the online GP update, each residual component is bounded element-wise as

(22) $${\tilde{r}}_{k}=\mathrm{clip}\left(r_{k},-0.5,\;0.5\right).$$

The input-residual pair $\left(z_{k}^{GP},{\tilde{r}}_{k}\right)$ is then added to the bounded online GP dataset. The GP predicts six residual components independently using a shared squared-exponential kernel. Only a fixed number of recent samples is retained to limit the computational cost of online GP inference and refitting. The six-dimensional residual function is modeled using a GP,

(23) $$r\left(z\right)∼GP\left(0,k\left(z,z^{\prime }\right)\right),$$

where $k\left(\cdot ,\cdot \right)$ denotes the covariance function. A shared squared-exponential radial basis function (RBF) kernel is used,

(24) $$k\left(z_{i},z_{j}\right)=\sigma_{f}^{2}\exp\left[-\frac{1}{2}{\left(z_{i}-z_{j}\right)}^{T}\Lambda^{-1}\left(z_{i}-z_{j}\right)\right],$$

where $\sigma_{f}^{2}$ is the signal variance and $\Lambda =l^{2}I$ represents the isotropic kernel length-scale matrix. The GP input and output data are standardized using the statistics of the currently retained online dataset. Six residual components are predicted independently using the shared kernel hyperparameters. During locomotion, newly observed input-residual pairs are appended to the GP dataset. To bound the computational cost, at most 200 recent samples are retained, and GP refitting is performed every five updates after a minimum of 12 samples has been collected. The kernel and residual-correction parameters used in the experiments are summarized in Section 3.2. For a query input $z_{k}^{GP}$, the GP provides the posterior residual mean

(25) $$\mu_{k}^{GP}={\left[\mu_{vx,k},\mu_{vy,k},\mu_{vz,k},\mu_{\omega x,k},\mu_{\omega y,k},\mu_{\omega z,k}\right]}^{T}$$

and the corresponding predictive standard deviation $\sigma_{k}^{GP}$. The predictive uncertainty is not directly included in the MPC objective or constraints. Instead, it is used to attenuate the residual correction through the confidence factor

(26) $$c_{k}=\exp\left(-2{\bar{\sigma }}_{k}^{GP}\right),{\bar{\sigma }}_{k}^{GP}=\frac{1}{6}\sum_{j=1}^{6}\sigma_{j,k}^{GP}.$$

The confidence-weighted GP residual is then computed as

(27) $$\Delta x_{k}^{GP}=\mathrm{clip}\left({1.5,c}_{k}\;\mu_{k}^{GP},-0.15,0.15\right).$$

Although the GP estimates all six velocity residual components, only the longitudinal component is applied to the MPC prediction in the present implementation. Therefore,

(28) $$\Delta x_{k,MPC}^{GP}=e_{vx}\Delta v_{x,k}^{GP},e_{vx}={\left[1,0,0,0,0,0\right]}^{T}.$$

This design restricts the learned correction to the forward-velocity channel while retaining the remaining nominal velocity dynamics in the MPC prediction. The resulting residual correction is incorporated into the finite-horizon command-level MPC described in Section 2.5.

### 2.5. GP-MPC Command Supervisor

The GP residual model described in Section 2.4 is incorporated into a command-level MPC framework. Rather than directly optimizing joint torques or contact forces, the proposed supervisor optimizes the velocity command supplied to the frozen PPO locomotion policy. The optimization variable is $u_{k+i|k}={\left[\begin{array}{ccc} v_{x,k+i|k}^{cmd} & v_{y,k+i|k}^{cmd} & \omega_{z,k+i|k}^{cmd} \end{array}\right]}^{T},$ where the lateral reference command is set to zero in the experiment.

This hierarchical design preserves the frozen PPO policy while allowing the high-level velocity command to be adjusted through predictive optimization [21,22,23,24].

**Figure 5:**
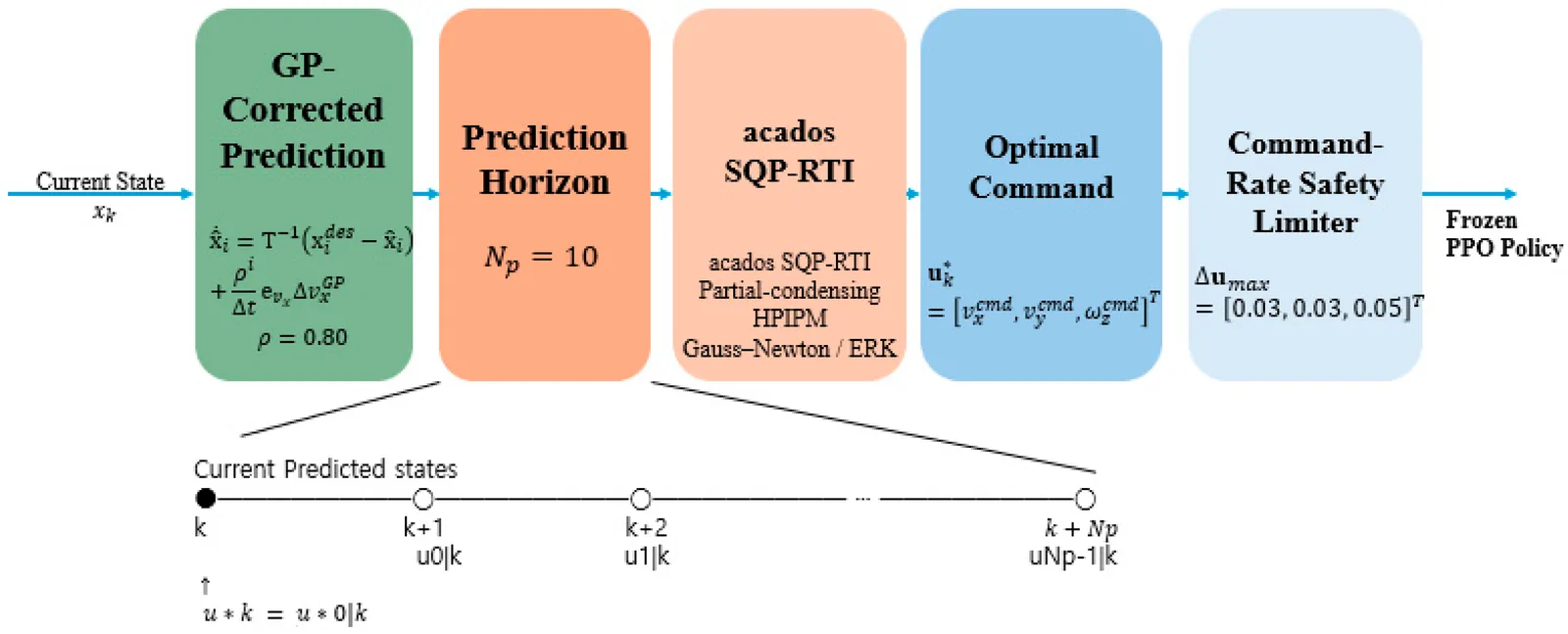
GP-MPC command supervisor. The online GP residual augments the nominal command-response prediction used by the finite-horizon MPC. Arrows indicate the sequence of prediction, optimization, command limiting, and command delivery to the frozen PPO policy.

As illustrated in Figure 5, the current robot state is first evaluated using the GP-corrected prediction model. At each supervisory update, the current six-dimensional velocity state is used as the initial condition of the prediction model. For prediction stage i, the desired velocity state associated with the optimized command is

(29) $$x_{k+i|k}^{des}={\left[\begin{array}{cccccc} v_{x,k+i|k}^{cmd}, & v_{y,k+i|k}^{cmd}, & 0, & 0, & 0, & \omega_{z,k+i|k}^{cmd} \end{array}\right]}^{T}.$$

The GP-augmented prediction dynamics are expressed as

(30) $${\dot{\hat{x}}}_{k+i|k}=T^{-1}\left(x_{k+i|k}^{des}-{\hat{x}}_{k+i|k}\right)+\frac{\rho^{i}}{\Delta t}e_{vx}\Delta v_{x,k}^{GP},$$

where $T=diag\left(0.24,0.30,0.18,0.20,0.20,0.22\right),$ Δt denotes the supervisory sampling interval, and $e_{v_{x}}={\left[1,0,0,0,0,0\right]}^{T},\;$ because the GP predicts a one-step residual, its contribution is gradually attenuated over the prediction horizon using

(31) $$\rho^{i}\Delta v_{x,k}^{GP},\rho =0.80.$$

Thus, the largest GP correction is applied to the immediate prediction stage, while its influence decreases at later stages. This avoids treating a one-step learned residual as a constant disturbance over the entire prediction horizon. Using the predicted velocity trajectory, the MPC determines an optimal command sequence by minimizing

(32) $$J\left(U\right)=\sum_{i=0}^{N_{p}-1}\left[{∥{\hat{x}}_{k+i|k}-x_{k+i}^{ref}∥}_{Q}^{2}+{∥u_{k+i|k}-u_{k+i}^{ref}∥}_{R}^{2}\right]+{∥{\hat{x}}_{k+N_{p}|k}-x_{k+N_{p}}^{ref}∥}_{Q_{f}}^{2},$$

where Q, R, and $Q_{f}$ denote the state-tracking, command-deviation, and terminal-state weighting matrices, respectively. The reference velocity state is defined as

(33) $$x^{ref}={\left[\begin{array}{cccccc} v_{x}^{ref}, & v_{y}^{ref}, & 0, & 0, & 0, & \omega_{z}^{ref} \end{array}\right]}^{T},$$

and the corresponding reference command is

(34) $$u^{ref}={\left[\begin{array}{ccc} v_{x}^{ref}, & v_{y}^{ref}, & \omega_{z}^{ref} \end{array}\right]}^{T}.$$

The command sequence is constrained by

(35) $$u_{min}\leq u_{k+i|k}\leq u_{max},$$

and the optimal sequence is obtained as

(36) $$U^{*}=\arg\;\underset{U}{min}J\left(U\right).$$

Following the receding-horizon strategy, only the first element of the optimal command sequence is selected,

(37) $$u_{k}^{*}=u_{0|k}^{*}.\;$$

The state-tracking, command-deviation, and terminal weighting matrices used in the experiments were

(38) Q=diag(25, 10, 5, 8, 8, 12),

(39) R=diag(5, 5, 3),

and

(40) $$Q_{f}=4Q.$$

The state weights emphasize forward-velocity tracking while penalizing undesired lateral, vertical, and angular velocity deviations. The command penalty limits unnecessary deviation from the reference command, while the larger terminal weight promotes tracking toward the end of the prediction horizon. The optimized command was constrained within

(41) $$u_{min}={\left[0,-0.15,-0.8\right]}^{T},u_{max}={\left[0.8,0.15,0.8\right]}^{T},$$

corresponding to forward-velocity, lateral-velocity, and yaw-rate command limits in units of m/s, m/s, and rad/s, respectively. These are the actual OCP command bounds used in the implementation. After the first optimized command was obtained, an additional command-rate safety limiter was applied before the command was supplied to the frozen PPO policy,

(42) $$u_{k}^{applied}=u_{k-1}^{applied}+\mathrm{clip}\left(u_{k}^{*},-u_{k-1}^{applied}-\Delta u_{max},\Delta u_{max}\right),$$

where

(43) $$\Delta u_{max}={\left[0.03,0.03,0.05\right]}^{T}.\;$$

Thus, the maximum changes per supervisory update were 0.03 m/s for the forward and lateral velocity commands and 0.05 rad/s for the yaw-rate command. This limiter was applied after the MPC optimization as a safety mechanism and was not included as an additional stage cost or OCP constraint.

The prediction horizon was set to $N_{p}=10$. The nonlinear optimal control problem was solved online using the acados framework with the Sequential Quadratic Programming Real-Time Iteration (SQP-RTI) scheme. The implementation employed the partially condensing HPIPM QP solver, a Gauss–Newton Hessian approximation, and explicit Runge–Kutta integration. One SQP-RTI step was executed at each supervisory update, with QP warm starting enabled and a Levenberg–Marquardt regularization coefficient of $1\times 10^{-6}$. These settings correspond directly to the numerical solver configuration used in the experiments. The frozen PPO locomotion policy, inverse-kinematics module, and low-level PD controller were identical for PPO, MPC-RL, and GP-MPC-RL. Therefore, the differences among the compared methods resulted from the command-level supervisory strategy.

## 3. Experimental Setup and Evaluation Protocol

This section describes the simulation environment, controller configuration, experimental scenarios, and evaluation metrics used to evaluate the proposed GP-MPC command-supervision framework. All experiments were conducted in Isaac Sim using a Unitree Go2 quadruped robot under identical low-level locomotion settings. To ensure a fair comparison, the PPO locomotion policy, inverse kinematics, and PD controller remained unchanged throughout all experiments. Only the command-level supervisor differed among the compared methods.

### 3.1. Simulation Environment

All experiments were performed in Isaac Sim 5.1 using the NVIDIA PhysX physics engine (NVIDIA, Santa Clara, CA, USA). The robot model was based on the Unitree Go2 quadruped platform, which consists of four identical legs with three actuated joints per leg, resulting in a total of twelve actuated joints. The simulator was executed at 200 Hz, while the low-level controller operated at 50 Hz. The GP-MPC supervisor was updated at the supervisory control rate specified in Section 3.2. To evaluate controller performance across different terrain conditions, four representative terrain types were considered: flat ground, gravel terrain, inclined slopes, and step obstacles. Each terrain was procedurally generated in Isaac Sim while maintaining identical environmental conditions across all compared methods. The main simulation and control settings are summarized in Table 1.

The random uneven terrain was procedurally generated using uniformly distributed height perturbations within ±0.05 m and a grid resolution of 0.10 m. Different random seeds were used to generate independent terrain realizations across trials, while the same set of terrain seeds was used for all compared controllers to enable paired performance comparisons.

### 3.2. Controller Configuration

The control system consisted of a frozen PPO locomotion policy, a GP residual model, and a command-level GP-MPC supervisor. The same PPO checkpoint, observation normalization, action limits, inverse kinematics solver, and PD gains were used for all comparative methods. Therefore, differences among PPO, MPC-RL, and GP-MPC-RL were attributable to the command-level supervisory strategy.

The PPO policy was trained offline in domain-randomized simulation environments. It received proprioceptive observations together with the commanded forward velocity and yaw rate, and generated the desired low-level locomotion actions. After training, the policy parameters were fixed and were not updated during the GP and MPC experiments. The main PPO training parameters are summarized in Table 2.

The discount factor and GAE parameter were selected to account for long-term locomotion performance while limiting the variance of the advantage estimate. The clipping parameter restricted excessively large policy updates during training, while the entropy term encouraged action exploration. The learning rate, number of epochs, and mini-batch size were selected within ranges commonly used for continuous-control locomotion tasks.

The GP residual model used a zero-mean prior and a squared exponential RBF kernel. The implemented multi-output GP receives a 19-dimensional input and predicts a six-dimensional residual vector. During locomotion, newly observed one-step prediction residuals are incorporated online into a bounded GP dataset, allowing the residual model to adapt while preventing unbounded growth of the online learning problem. The GP predictive uncertainty is not directly included in the MPC objective function or constraints; instead, it is used to attenuate the residual correction through a confidence factor. In the present implementation, only the forward-velocity residual component is applied to the MPC prediction. The GP hyperparameters and residual-correction settings used in the experiments are summarized in Table 3.

The GP correction was evaluated online before each supervisory optimization step. All GP hyperparameters, dataset bounds, residual-correction limits, MPC weights, and command constraints were kept fixed across the comparative experiments. No controller-specific retuning was performed for different target velocities or payload conditions. At each supervisory update, the previously obtained optimal solution was reused as an initial guess for the next SQP-RTI problem. Only the first optimized command was applied to the frozen PPO policy according to the receding-horizon principle. To evaluate the contribution of command-level predictive supervision and online GP residual correction, three controller configurations were compared. All methods used the same frozen PPO locomotion policy, inverse-kinematics solver, PD controller, robot model, and simulation settings. Therefore, the compared methods differed only in the command-level supervisory component. The controller configurations are summarized in Table 4.

The comparison isolates the respective contributions of command-level MPC supervision and online bounded GP residual correction by progressively introducing the MPC supervisor and the GP-corrected prediction model while keeping all other controller components unchanged.

### 3.3. Experimental Conditions and Evaluation Protocol

To comprehensively evaluate the proposed GP-MPC command supervision framework, four representative locomotion scenarios were designed to investigate its performance under progressively challenging terrain conditions. Each scenario was intended to evaluate a different aspect of command-level locomotion control, including velocity tracking, body stabilization, terrain adaptability, and obstacle traversal.

For all experiments, the same frozen PPO locomotion policy, inverse kinematics solver, and PD controller were used. Consequently, differences in locomotion performance were associated with the command-level supervision strategy. Each controller was evaluated over the same set of twenty independently generated terrain trials, and the reported results correspond to the mean and standard deviation across all trials.

The compared methods were defined as follows.

- PPO: Frozen PPO locomotion policy without command supervision.
- MPC + RL: Command-level MPC using only the nominal command-response model.
- GP-MPC + RL: Command-level MPC using the GP-corrected prediction model proposed in this work.

**Figure 6:**
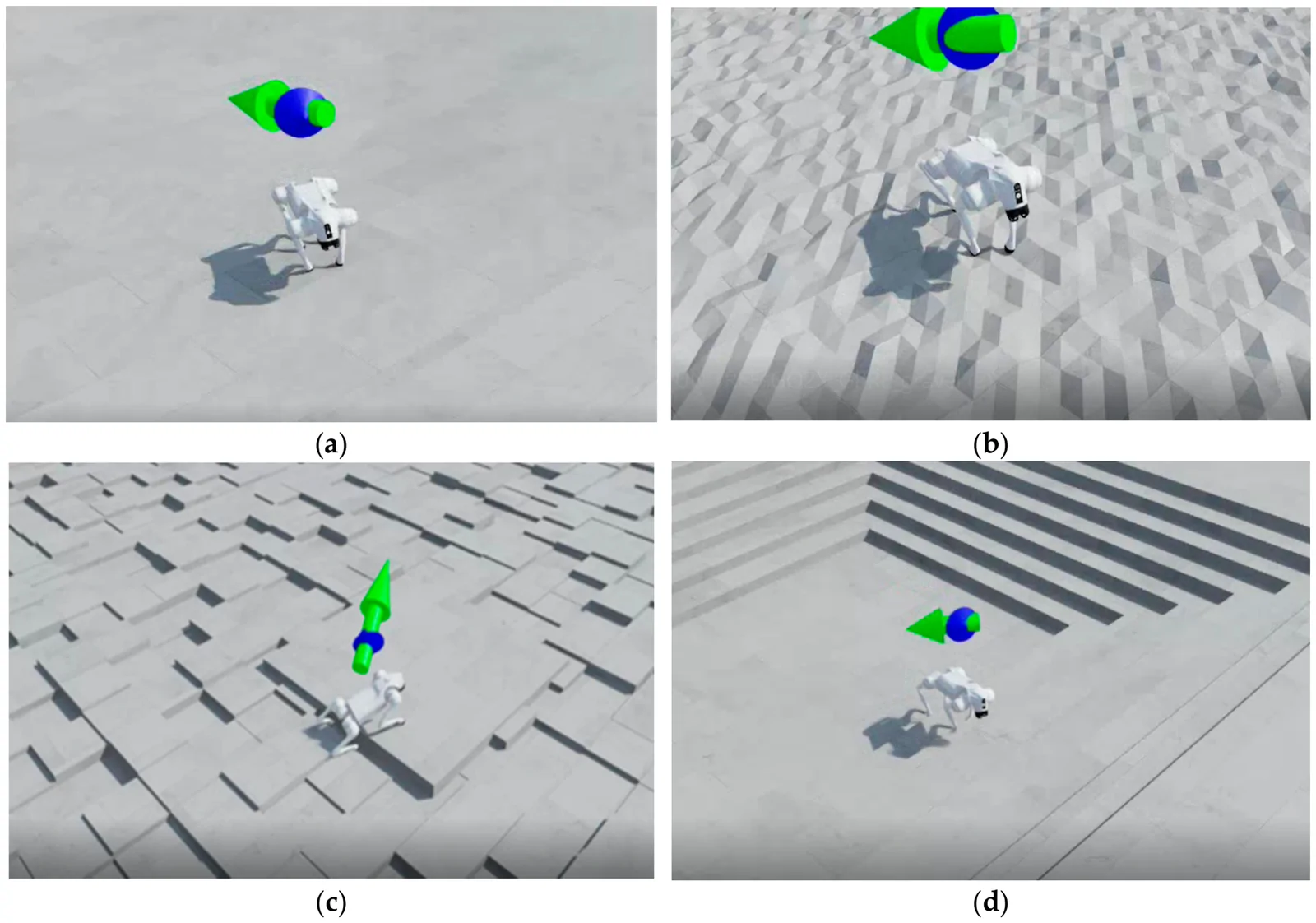
Representative terrain scenarios used in the simulation study: (**a**) flat ground, (**b**) procedurally generated random uneven terrain, (**c**) a 15° uphill slope, and (**d**) 10 cm step obstacles. Colors in the screenshots are used only for simulation visualization and do not represent quantitative variables.

Representative terrain environments considered in the simulation framework are illustrated in Figure 6 and summarized in Table 5. The main quantitative controller comparisons reported in Section 5 were conducted on procedurally generated rough terrain using 20 paired trials per operating condition. Additional terrain types were used to evaluate the broader applicability of the framework and the terrain-conditioned formulation described in Section 4.

Scenario 1: Flat Ground The first experiment was conducted on flat terrain to establish the baseline locomotion performance under nominal operating conditions. Since external terrain disturbances were negligible, this scenario was used to evaluate velocity-tracking accuracy and body stability without significant modeling uncertainty.

Scenario 2: Random Uneven Terrain The second experiment evaluated locomotion over procedurally generated random uneven terrain. The terrain was generated by randomly varying the ground height within $-0.05\;m\leq h\left(x,y\right)\leq 0.05\;m,\;$ using a grid resolution of 0.10 m. Different random seeds were used across trials to generate independent terrain realizations, while the same terrain-seed set was used for all compared controllers. Unlike structured environments, the robot had no prior knowledge of the terrain geometry, resulting in continuously changing foot–ground contact conditions and larger potential model mismatch. This scenario was designed to evaluate whether the proposed GP-MPC supervisor could compensate for terrain-dependent modeling errors while maintaining command-tracking performance under irregular surface conditions.

Scenario 3: Uphill Slope The third experiment evaluated locomotion on a 15° uphill slope. Compared with flat terrain, the robot continuously experienced gravitational disturbances that affected body posture and forward velocity. The purpose of this experiment was to evaluate the ability of the proposed controller to maintain roll and pitch stability while accurately tracking the commanded velocity.

Scenario 4: Step Obstacle The final experiment consisted of repeated traversal over 10 cm step obstacles. Unlike the previous scenarios, the robot experienced abrupt changes in contact conditions during each stepping motion, producing relatively large prediction errors in the nominal model. This scenario evaluated the performance of the proposed GP-MPC supervisor during obstacle negotiation and investigated its ability to generate smooth velocity commands under rapidly changing contact conditions. The performance of all compared controllers was evaluated using the quantitative metrics described in Section 3.4, including velocity-tracking error, body-attitude error, command smoothness, and locomotion success rate. For the quantitative controller comparisons, the results are reported as the mean and standard deviation over 20 trials per operating condition, using the same set of random seeds across the compared controllers to enable paired analysis.

### 3.4. Evaluation Metrics

Accurate velocity tracking and body-attitude regulation are both important for stable quadruped locomotion. Persistent forward-velocity errors can cause deviations from the commanded locomotion behavior, whereas excessive roll and pitch motion can alter foot-loading distribution and reduce the stability margin during uneven-terrain contact. Therefore, velocity tracking and body-attitude regulation are evaluated jointly in this study rather than treating tracking accuracy alone as sufficient locomotion performance. The proposed GP-MPC command-supervision framework was evaluated using forward-velocity tracking error, body-attitude error, command smoothness, longitudinal model residual, and supervisory computation time. These metrics characterize tracking accuracy, body-attitude regulation, command continuity, model mismatch, and computational performance, respectively [18,19,24,25]. Unless otherwise stated, the controller-comparison results in Section 5 are reported as the mean and standard deviation over 20 trials per operating condition.

#### 3.4.1. Velocity Tracking Error

The forward velocity tracking performance was quantified using the root mean square error (RMSE), which is widely used to evaluate the deviation between the commanded and measured velocities [24].

(44) $$RMSE_{v}=\sqrt{\frac{1}{N}\sum_{k=1}^{N}{\left(v_{x,k}-v_{x,k}^{ref}\right)}^{2}},$$

where $v_{x,k}$ and $v_{x,k}^{ref}$ denote the measured and commanded forward velocities, respectively, and N is the total number of control samples.

#### 3.4.2. Body-Attitude Error

Robot stability was evaluated using the RMSE of the body roll and pitch angles, which are commonly used indicators of locomotion stability on uneven terrain [21,22].

(45) $$RMSE_{\phi }=\sqrt{\frac{1}{N}\sum_{k=1}^{N}(\left.\phi_{k}-\phi_{k}^{ref}{\left.)\right.}^{2}\right.},$$

(46) $$RMSE_{\theta }=\sqrt{\frac{1}{N}\sum_{k=1}^{N}(\left.\theta_{k}-\theta_{k}^{ref}{\left.)\right.}^{2}\right.},$$

where ϕ and θ denote the body roll and pitch angles, respectively. Since the desired body posture corresponds to level locomotion,

(47) $$\phi^{ref}=\theta^{ref}=0.$$

Lower RMSE values indicate smaller body-attitude deviations during locomotion.

#### 3.4.3. Command Smoothness

The smoothness of the supervisory command was evaluated by the accumulated command variation,

(48) $$CS=\sum_{k=2}^{N}∥u_{k}-u_{k-1}{∥}_{2}^{2},$$

where

(49) $$u_{k}=\left[\begin{array}{c} v_{x}^{cmd}\\ \omega_{z}^{cmd} \end{array}\right].$$

This metric penalizes abrupt changes in successive commands and is frequently adopted in predictive control to evaluate command continuity [21,23]. Lower values indicate smoother command generation.

#### 3.4.4. Longitudinal Model Residual

The longitudinal model mismatch was quantified using the one-step forward-velocity residual between the measured robot response and the nominal model prediction,

(50) $$r_{vx,k}=v_{x,k+1}^{meas}-{\hat{v}}_{x,k+1}^{nom}.$$

Because positive and negative residuals may cancel when averaged directly, the residual magnitude was evaluated using the mean absolute longitudinal residual,

(51) $$\bar{|r_{vx}|}=\frac{1}{N}\sum_{k=1}^{N}|r_{v_{x},k}|.$$

A larger value indicates a greater discrepancy between the nominal command-response prediction and the measured robot dynamics.

#### 3.4.5. Supervisor Computation-Time Metric

Since the proposed framework is intended for real-time command supervision, its computational efficiency was evaluated using the average supervisor-level computation time per update,

(52) $$t_{avg}=\frac{1}{N}\sum_{k=1}^{N}t_{k},$$

where $t_{k}$ denotes the computation time required to solve the GP-MPC optimization problem at the k-th control step. The timing was measured on the same computing platform for all compared supervisory methods. The reported values quantify only the supervisor-level computation and do not include the complete end-to-end latency associated with sensing, PPO-policy inference, communication, low-level control, physics simulation, or hardware execution. Therefore, the timing results are used to characterize the computational overhead of the supervisor rather than the complete real-time system latency [21,23]. Unless otherwise stated, the reported values correspond to the mean ± standard deviation over 20 trials per operating condition, with the same random seeds used across the compared controllers for paired analysis. Normality of the paired differences was assessed using the Shapiro–Wilk test. A paired *t*-test was used for approximately normally distributed paired differences, whereas the Wilcoxon signed-rank test was used when the normality assumption was violated. Statistical significance was defined as p<0.05 [25]. During a representative 50-update solver diagnostic, all optimization calls returned a successful solver status (status = 0), with 4.58 ± 1.01 QP iterations per update (range: 3–8). Because SQP-RTI performs a single real-time iteration at each control update, these results are reported as numerical solver behavior rather than as proof of asymptotic SQP convergence.

## 4. Additional Terrain-Conditioned GP-MPC Implementation and Validation

### 4.1. Implementation Overview

This terrain-conditioned implementation is a separate additional validation and is not the online bounded GP controller used for the main PPO, MPC-RL, and GP-MPC-RL comparisons in Section 5. As an additional validation, a terrain-conditioned variant of the GP-MPC framework was implemented by explicitly incorporating continuous local-terrain features into the residual model. Unlike terrain-label-based supervision, this additional implementation does not assign the ground surface to a predefined terrain category during control. Instead, continuous geometric information is extracted directly from a local height map and incorporated into the residual dynamics model.

At each control instant, the current robot state and the supervisory control input are combined with a four-dimensional terrain descriptor. The terrain descriptor consists of the local height, longitudinal inclination, lateral inclination, and local surface roughness. These variables are supplied to a GP model that estimates the discrepancy between the nominal state transition and the terrain-affected state transition. The estimated residual is then used to correct the nominal prediction employed by the model predictive controller. The finite-horizon optimal control problem is constructed in CasADi and solved using the acados framework. Only the first control input of the optimized sequence is applied, after which the state and terrain information are updated and the optimization is repeated.

**Figure 7:**
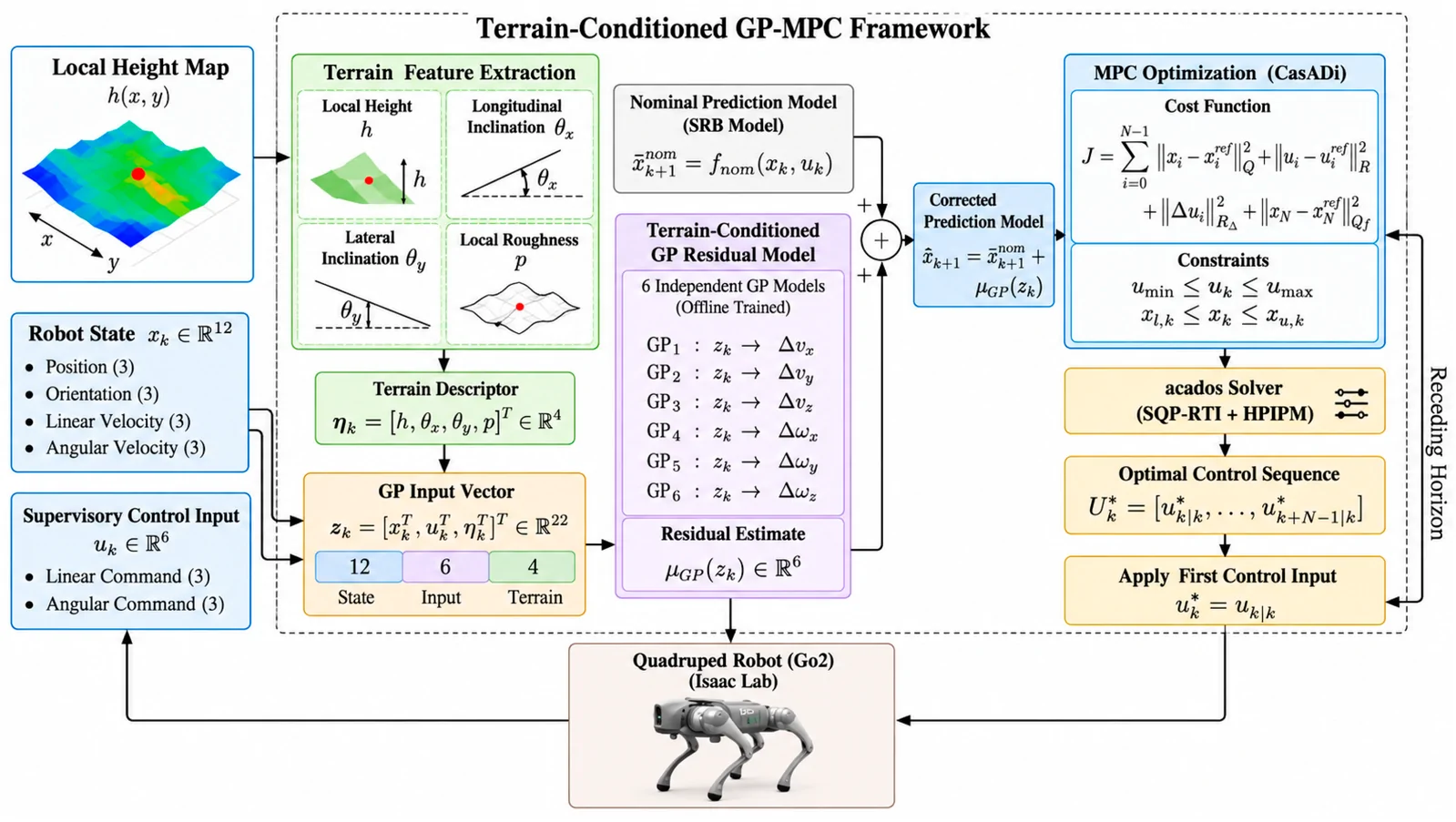
Overall implementation architecture of the terrain-conditioned GP-MPC framework. Colors group the terrain, GP, prediction, and optimization modules, while arrows indicate information and control flow.

This implementation therefore separates the control process into three functional stages. First, the local terrain geometry is represented using continuous features. Second, the terrain-dependent transition residual is estimated from the current state, input, and terrain descriptor. Third, the corrected prediction model is used in a receding-horizon optimization procedure. The complete terrain-conditioned control architecture is shown in Figure 7.

### 4.2. Continuous Terrain Representation

**Figure 8:**
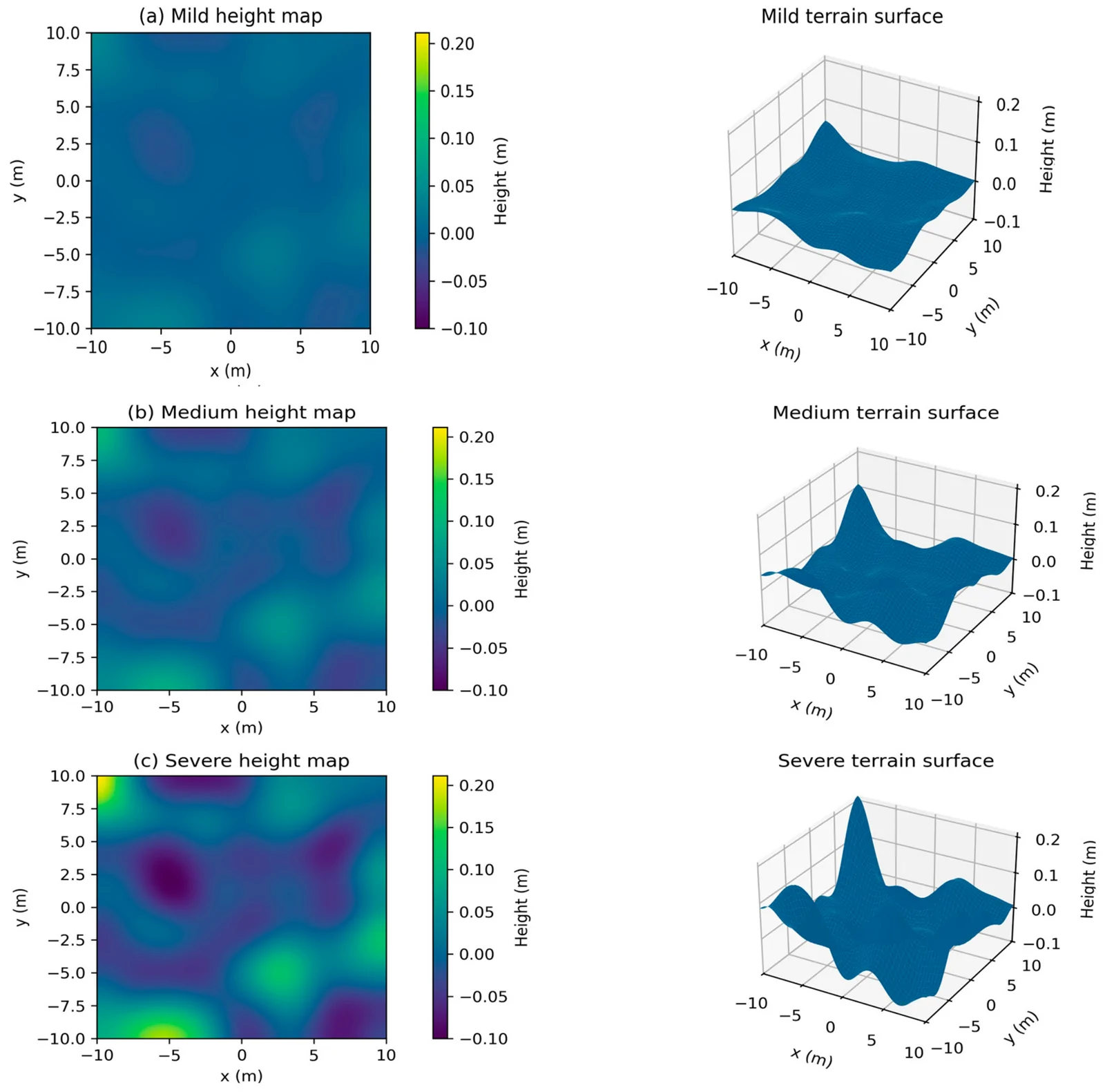
Generated height maps for the three terrain-severity levels: (**a**) mild, (**b**) medium, and (**c**) severe.

Three uneven-terrain levels were generated using smoothed random height fields. The terrain levels were designed to provide progressively larger height variations while avoiding unrealistic discontinuities and excessively steep local gradients. Gaussian filtering was applied to the initially sampled random field, after which the terrain height was normalized to the prescribed standard deviation. The mild, medium, and severe terrains were generated with height standard deviations of 0.010, 0.025, and 0.050 m, respectively. The measured maximum slopes were approximately 2.09°, 5.21°, and 10.34°. These terrain conditions were used to investigate whether controller performance degraded gradually as the geometric difficulty increased. Representative height maps for the three terrain-severity levels are shown in Figure 8.

For a local ground position $\left(x_{f},y_{f}\right)$, the terrain descriptor $\eta_{k}\in R^{4}$ was defined as

(53) $$\eta_{k}={\left[\begin{array}{cccc} h_{k} & \theta_{x,k} & \theta_{y,k} & \rho_{k} \end{array}\right]}^{⊤},$$

where $h_{k}$ is the local height, $\theta_{x,k}$ and $\theta_{y,k}$ are the longitudinal and lateral inclination angles, and $\rho_{k}$ denotes the local roughness.

The inclination angles were calculated using the height gradients:

(54) $$\theta_{x,k}={tan}^{-1}\left(\frac{\partial h}{\partial x}\right),\;\theta_{y,k}={tan}^{-1}\left(\frac{\partial h}{\partial y}\right).$$

The local roughness was calculated from the standard deviation of the height samples inside a neighborhood surrounding the query position:

(55) $$\rho_{k}=\sqrt{\frac{1}{N_{p}}\sum_{j=1}^{N_{p}}{\left(h_{j}-\bar{h}\right)}^{2}}.$$

Unlike a discrete terrain identifier, $\eta_{k}$ changes continuously with the local ground geometry. It can therefore represent intermediate terrain conditions and transitions that cannot be expressed using a fixed set of semantic labels.

Table 6 confirms that the terrain generator increased both the height variation and the maximum local inclination in a controlled manner. The Gaussian smoothing procedure prevented the local slope from increasing unrealistically despite the larger elevation range of the severe terrain.

### 4.3. Terrain-Conditioned Residual Dataset

For the additional terrain-conditioned validation, a dedicated transition dataset was constructed to train the terrain-conditioned GP model. As shown in Figure 8, each sample was generated by evaluating the nominal dynamics and the terrain-affected dynamics from the same initial state and control input. The target value was not defined as the external terrain force itself. Instead, the GP was trained using the corresponding one-step state-transition discrepancy. This definition ensured consistency between the learned output and the residual correction applied to the predictive model.

The nominal one-step transition is denoted by

(56) $$x_{k+1}^{nom}=f_{nom}\left(x_{k},u_{k}\right).$$

The terrain-affected transition is represented by

(57) $$x_{k+1}^{ter}=f_{ter}\left(x_{k},u_{k},\eta_{k}\right).$$

The learning target was defined using the linear- and angular-velocity components:

(58) $$r_{k}=x_{k+1,7:12}^{ter}-x_{k+1,7:12}^{nom},$$

with

(59) $$r_{k}={\left[\begin{array}{cccccc} \Delta v_{x} & \Delta v_{y} & \Delta v_{z} & \Delta \omega_{x} & \Delta \omega_{y} & \Delta \omega_{z} \end{array}\right]}^{⊤}.$$

The GP input was constructed as

(60) $$z_{k}^{TC}={\left[\begin{array}{ccc} x_{k}^{⊤} & u_{k}^{⊤} & \eta_{k}^{⊤} \end{array}\right]}^{⊤}\in R^{22}.$$

The 22-dimensional input consists of a 12-dimensional robot state, a six-dimensional control input, and a four-dimensional continuous terrain descriptor. A total of 5000 state–input–terrain samples were generated.

**Figure 9:**
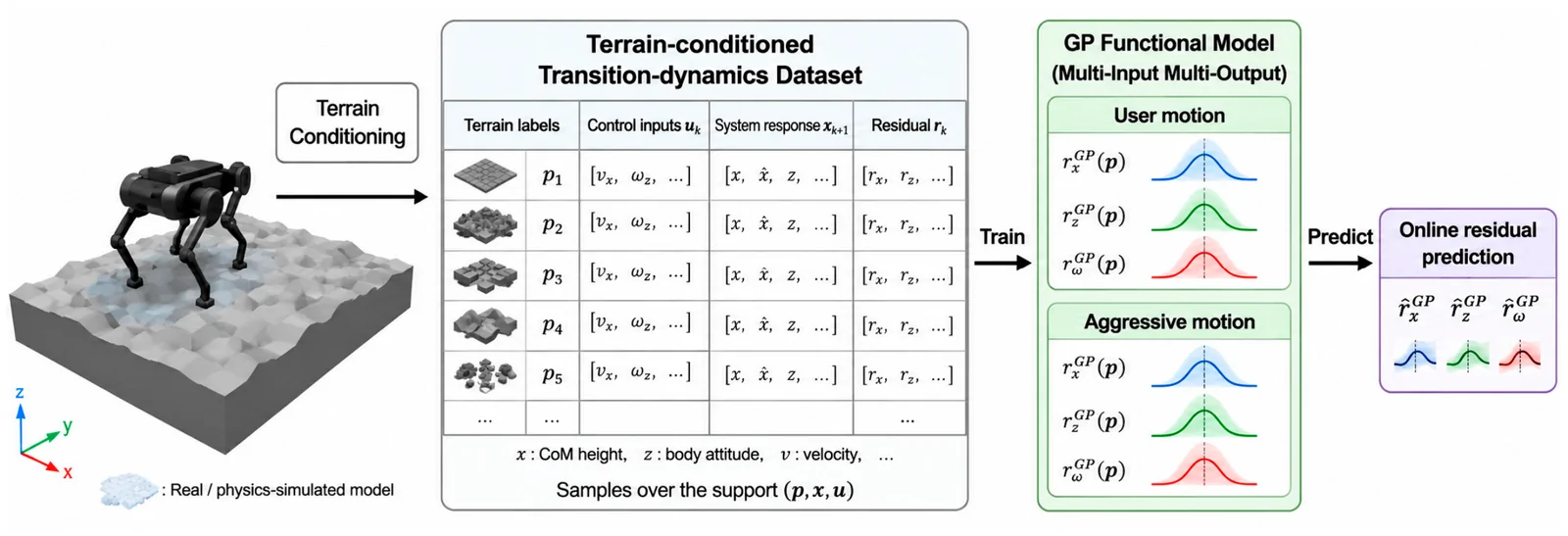
Procedure used to construct the terrain-conditioned transition-residual dataset. Arrows indicate the flow from terrain conditioning and dataset construction to GP training and one-step residual prediction.

The simulated contact location was restricted to a quadruped-scale support region to avoid unrealistically large body moments. The terrain-induced force and moment were converted to changes in linear and angular velocity using the body mass, inertia, and sampling interval. This process produced transition residuals with physically interpretable units that could be added directly to the nominal one-step state prediction. The corresponding transition-residual dataset construction procedure is summarized in Figure 9.

For this additional validation, a dedicated terrain-conditioned dataset was generated to train the GP residual model. Each data sample consists of the current robot state, the corresponding control input, and the local terrain descriptor extracted from the surrounding height map. The terrain descriptor includes the local terrain height, longitudinal slope, lateral slope, and surface roughness, enabling the GP model to capture the influence of terrain geometry on the robot dynamics.

The input vector has a total dimension of 22, composed of a 12-dimensional robot state, a 6-dimensional control input, and a 4-dimensional terrain feature vector. The learning target is defined as the one-step residual between the nominal and terrain-affected state transitions, represented by the linear and angular velocity components. Consequently, the GP predicts a six-dimensional residual vector rather than the external disturbance itself.

To ensure sufficient coverage of various operating conditions, a total of 5000 state–input–terrain samples were generated through repeated simulations over terrains with different severity levels. The generated dataset provides representative terrain-dependent transition errors that are subsequently used to train six independent GP regressors.

As shown in Table 7, the terrain interaction predominantly affected the vertical and body-angular responses. The residuals in the horizontal velocity components remained comparatively small, whereas the roll- and pitch-rate residuals exhibited wider distributions because of terrain-induced body moments.

### 4.4. Gaussian-Process Residual Estimation

For this terrain-conditioned validation, six independent GP regressors were trained to estimate the six components of the transition residual. The covariance function consisted of a constant kernel, a radial basis function kernel, and a white-noise term:

(61) $$k\left(z^{TC},{z^{TC}}^{\prime }\right)=\sigma_{f}^{2}\exp\left[-\frac{1}{2}{\left(z^{TC}-{z^{TC}}^{\prime }\right)}^{⊤}\Lambda^{-1}\left(z^{TC}-{z^{TC}}^{\prime }\right)\right]+\sigma_{n}^{2}\delta_{z,z^{\prime }}.$$

Here, Λ contains the input-dependent length scales, $\sigma_{f}^{2}$ is the signal variance, and $\sigma_{n}^{2}$ represents measurement and modeling noise.

For a query input $z_{*}$, the posterior mean for the j-th residual component is

(62) $$\mu_{j}\left(z_{*}\right)=k_{*}^{⊤}{\left(K+\sigma_{n}^{2}I\right)}^{-1}y_{j}.$$

The complete residual estimate is

(63) $$\mu_{GP}\left(z_{*}\right)={\left[\begin{array}{cccc} \mu_{1}\left(z_{*}\right) & \mu_{2}\left(z_{*}\right) & \cdots & \mu_{6}\left(z_{*}\right) \end{array}\right]}^{⊤}.$$

**Figure 10:**
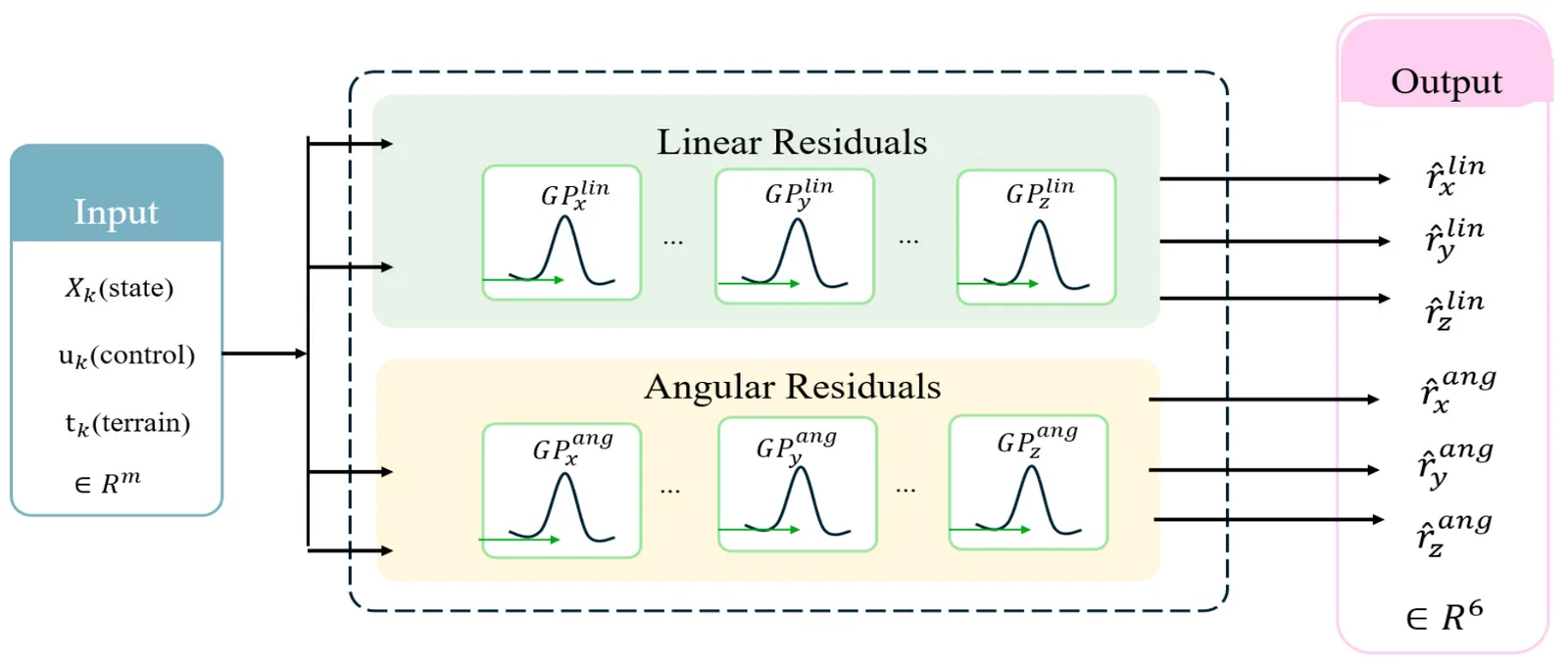
Six-output residual-estimation structure using six independent GP regressors. Arrows indicate the input-to-output prediction flow; ellipses denote repeated independent GP regressors for the remaining residual components.

The GP models were trained offline and kept fixed during the controller evaluation. This design isolates the effect of terrain conditioning from online model updates and ensures that all compared trials use the same learned residual model. The six-output residual-estimation structure is illustrated in Figure 10.

For this additional terrain-conditioned variant, the six GP models were trained offline and kept fixed during controller evaluation. This design isolates the effect of explicit terrain conditioning from online GP adaptation and ensures that all compared trials use the same terrain-conditioned residual models.

### 4.5. CasADi–Acados Predictive-Control Implementation

The optimal control problem was constructed symbolically using CasADi (version 3.7.2) and exported to acados for numerical solution. The predictive model contains 12 states and six control inputs. The state includes body position, orientation, linear velocity, and angular velocity, while the input represents the generalized linear and angular control quantities used by the supervisory model.

The terrain-conditioned prediction was defined as

(64) $${\hat{x}}_{k+1}=f_{nom}\left(x_{k},u_{k}\right)+B_{r}\mu_{GP}\left(x_{k},u_{k},\phi_{k}\right),$$

where $B_{r}$ maps the six-dimensional GP residual to the linear- and angular-velocity components of the 12-dimensional state.

The finite-horizon objective was expressed as

(65) $$J=\sum_{i=0}^{N-1}\left[{∥x_{i|k}-x_{i|k}^{ref}∥}_{Q}^{2}+{∥u_{i|k}-u_{i|k}^{ref}∥}_{R}^{2}\right]+{∥x_{N|k}-x_{N|k}^{ref}∥}_{Q_{N}}^{2}.\;$$

A prediction horizon of ten steps and a sampling interval of 0.05 s were used, corresponding to a prediction window of 0.5 s. Input constraints were imposed component wise:

(66) $$u_{min}\leq u_{i|k}\leq u_{max}.$$

The nonlinear program was solved using the SQP-RTI method with the partially condensing HPIPM quadratic-programming solver. CasADi was used to generate the explicit dynamics and sensitivity functions required by acados. At every control cycle, the current state was imposed as the initial-state constraint, the state and input references were updated throughout the horizon, and the first optimized input was applied. The predictive-controller and solver configuration is summarized in Table 8.

### 4.6. Evaluation Protocol

For the additional terrain-conditioned validation, three controller configurations were compared under mild, medium, and severe terrain conditions. The nominal MPC used the uncorrected prediction model and was directly exposed to the terrain disturbance. The GP-MPC baseline applied a residual-compensation mechanism without using explicit local terrain descriptors. The terrain-conditioned GP-MPC used the 22-dimensional state–input–terrain vector to estimate the terrain-dependent transition residual. All controllers used the same prediction horizon, sampling interval, state reference, input limits, rollout duration, and random seeds. Each rollout consisted of 300 control steps. Performance was evaluated after the initial transient period using the following metrics:

(67) $${RMSE}_{v}=\sqrt{\frac{1}{N_{e}}\sum_{k=1}^{N_{e}}{\left(v_{x,k}-v_{x}^{ref}\right)}^{2}},$$

$${RMSE}_{\phi }=\sqrt{\frac{1}{N_{e}}\sum_{k=1}^{N_{e}}\phi_{k}^{2}},{RMSE}_{\theta }=\sqrt{\frac{1}{N_{e}}\sum_{k=1}^{N_{e}}\theta_{k}^{2}}.\;$$

Control smoothness was defined from the variation between consecutive control vectors:

(68) $$S_{u}=\frac{1}{N-1}\sum_{k=1}^{N-1}{∥u_{k+1}-u_{k}∥}_{2}.$$

The velocity-tracking tolerance rate was calculated as the proportion of evaluation steps for which the absolute forward-velocity error remained below 0.1 m/s. Solver time was measured for every acados call and averaged over the rollout. The controller configurations used in this validation are summarized in Table 9.

### 4.7. Terrain-Severity Results

Table 10 presents the performance of the three controllers over the three terrain-severity levels.

The nominal MPC showed progressive deterioration as terrain severity increased. Its velocity RMSE increased from 0.0372 on mild terrain to 0.1006 on severe terrain, while the tracking tolerance rate decreased from 100% to 67.8%.

Both GP-based controllers maintained a 100% tracking tolerance rate under the medium and severe terrain conditions. The terrain-conditioned GP-MPC produced the lowest velocity RMSE at every terrain level. On severe terrain, it reduced the velocity RMSE by approximately 67.5% relative to nominal MPC and by approximately 10.4% relative to GP-MPC. The most pronounced improvement was observed in control smoothness. On severe terrain, the smoothness metric decreased from 0.7449 for nominal MPC and 0.0802 for GP-MPC to 0.0110 for terrain-conditioned GP-MPC. These results indicate that incorporating continuous terrain information was associated with lower command variation as terrain severity increased. However, the terrain-conditioned model did not produce the lowest attitude RMSE. GP-MPC achieved lower roll and pitch errors across all three terrain conditions. This result suggests that the current terrain-conditioned correction prioritizes forward-velocity tracking and command regularity, However, the terrain-conditioned GP-MPC did not produce the lowest body-attitude RMSE. GP-MPC achieved lower roll and pitch RMSE values across the tested terrain conditions, indicating that explicit terrain conditioning did not uniformly improve body-attitude regulation. Therefore, this additional validation should not be interpreted as demonstrating uniform superiority across all metrics. Rather, the principal benefits of the terrain-conditioned variant were improved forward-velocity tracking, a consistently high velocity-tracking tolerance rate, and reduced command variation as terrain severity increased.

## 5. Results and Discussion

The main controller-comparison experiments were conducted over 20 rough-terrain trials per operating condition. The same set of random seeds was used across PPO, MPC-RL, and GP-MPC-RL to enable paired statistical comparisons. For each trial index, the same terrain seed and initial robot configuration were used across PPO, MPC-RL, and GP-MPC-RL to enable paired comparisons. The reported results are expressed as the mean ± standard deviation over the twenty trials. Forward-velocity and body-attitude performance were evaluated using $RMSE_{v_{x}}$, $RMSE_{\phi }$, and $RMSE_{\theta }$, respectively.

### 5.1. Velocity Tracking Performance

The first experiment evaluated the forward-velocity tracking performance at target velocities of 0.3, 0.5, and 0.7 m/s. Table 11 summarizes the mean velocity RMSE for the three control configurations.

At 0.3 m/s, the mean RMSE decreased from 0.0666±0.0248 m/s for PPO to 0.0459±0.0215 m/s for MPC-RL and further to 0.0411±0.0109 m/s for GP-MPC-RL. This corresponds to a 38.3% reduction relative to PPO and a 10.4% reduction relative to MPC-RL. At 0.5 m/s, PPO produced an RMSE of 0.0750±0.0204 m/s, while MPC-RL and GP-MPC-RL achieved 0.0599±0.0144 m/s and 0.0590±0.0142 m/s, respectively.

Thus, both supervisory controllers clearly improved tracking relative to PPO, whereas the additional improvement provided by GP compensation over nominal MPC-RL was small under this nominal operating condition. At 0.7 m/s, the RMSE values were 0.0982±0.0424 m/s for PPO, 0.0943±0.0411 m/s for MPC-RL, and 0.0883±0.0349 m/s for GP-MPC-RL. GP-MPC-RL therefore reduced the mean error by 10.1% relative to PPO and 6.4% relative to MPC-RL. Because the paired RMSE differences were not normally distributed for most operating conditions, the Wilcoxon signed-rank test was used as the primary paired comparison. GP-MPC-RL significantly improved velocity tracking relative to PPO at 0.3 m/s (p<0.001), 0.5 m/s (p<0.001), and 0.7 m/s (p=0.0027).

**Figure 11:**
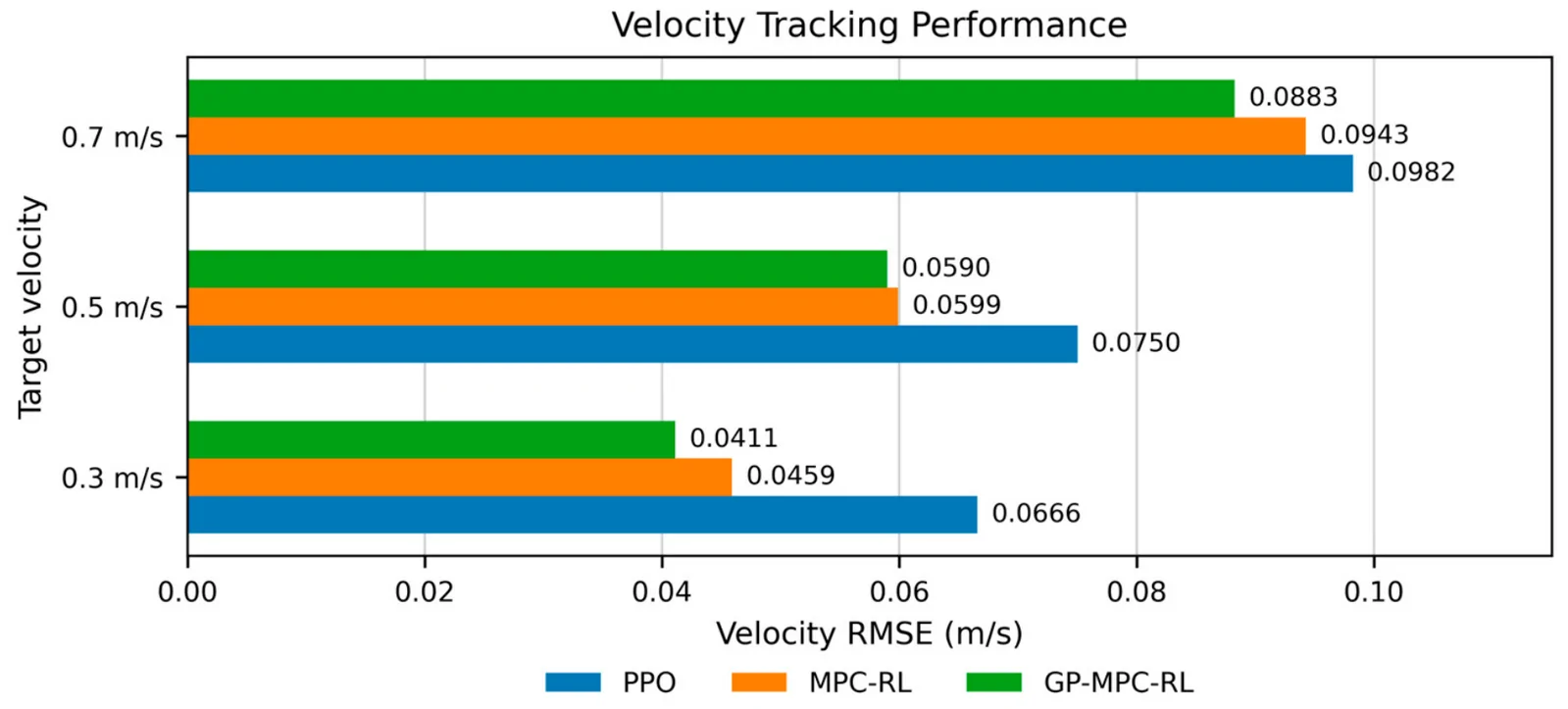
Forward-velocity tracking RMSE for PPO, MPC-RL, and GP-MPC-RL at target velocities of 0.3, 0.5, and 0.7 m/s. Each bar represents the mean over 20 trials.

Relative to MPC-RL, the additional GP correction was significant at 0.3 m/s (p=0.017) but not at 0.5 m/s (p=0.475) or 0.7 m/s (p=0.277). These results indicate that the MPC supervisory layer provides the majority of the improvement under nominal walking conditions, while the GP residual term provides an additional, but generally modest, benefit when the nominal command-response model already describes the locomotion response reasonably well. The forward-velocity tracking comparison is visualized in Figure 11.

### 5.2. Command Correction and Residual Dynamics

Table 12 summarizes the supervisory command modification, longitudinal model residual, and supervisor-level computation time. Command RMSE quantifies the difference between the target forward velocity and the command generated by the supervisory controller. For PPO, the target command is passed directly to the locomotion policy; therefore, its command RMSE is zero by definition and does not represent the actual velocity-tracking error.

For MPC-RL, the command RMSE values were 0.0397±0.0102, 0.0451±0.0129, and 0.0402±0.0117 m/s at target velocities of 0.3, 0.5, and 0.7 m/s, respectively. The corresponding GP-MPC-RL values were 0.0448±0.0102, 0.0487±0.0122, and 0.0425±0.0126 m/s. The slightly larger command RMSE of GP-MPC-RL under nominal conditions should not be interpreted as poorer tracking performance. Instead, it reflects the additional supervisory intervention introduced by the GP-corrected prediction model. As shown in Table 11, the resulting measured velocity RMSE remained comparable to or lower than that of MPC-RL at all three target velocities.

The magnitude of the measured longitudinal one-step residual, reported as the mean absolute residual $mean|r_{vx}|$, increased with target speed. For MPC-RL, the values were 0.01288±0.00315, 0.01541±0.00340, and 0.01873±0.00588 m/s at 0.3, 0.5, and 0.7 m/s, respectively. For GP-MPC-RL, the corresponding values were 0.01274±0.00251, 0.01551±0.00305, and 0.01846±0.00522 m/s.

The similarity of the residual magnitudes under the nominal 0 kg condition indicates that the nominal command-response model already represented the policy dynamics reasonably well in this operating range. Consequently, the main role of GP compensation is expected to become more evident when the nominal model is subjected to a larger dynamics mismatch, as investigated in Section 5.4.

### 5.3. Body-Attitude Regulation

Body-attitude stability was evaluated using roll and pitch RMSE, as summarized in Table 13.

At 0.3 m/s, PPO, MPC-RL, and GP-MPC-RL produced roll RMSE values of 0.0559 ± 0.0117, 0.0499 ± 0.0085, and 0.0512 ± 0.0084 rad, respectively. The corresponding pitch RMSE values were 0.1414 ± 0.0696, 0.1427 ± 0.0530, and 0.1496 ± 0.0631 rad.

At 0.5 m/s, PPO, MPC-RL, and GP-MPC-RL showed roll RMSE values of 0.0449 ± 0.0136, 0.0430 ± 0.0112, and 0.0414 ± 0.0091 rad, respectively, while the corresponding pitch RMSE values were 0.1560 ± 0.0763, 0.1549 ± 0.0729, and 0.1534 ± 0.0696 rad.

**Figure 12:**
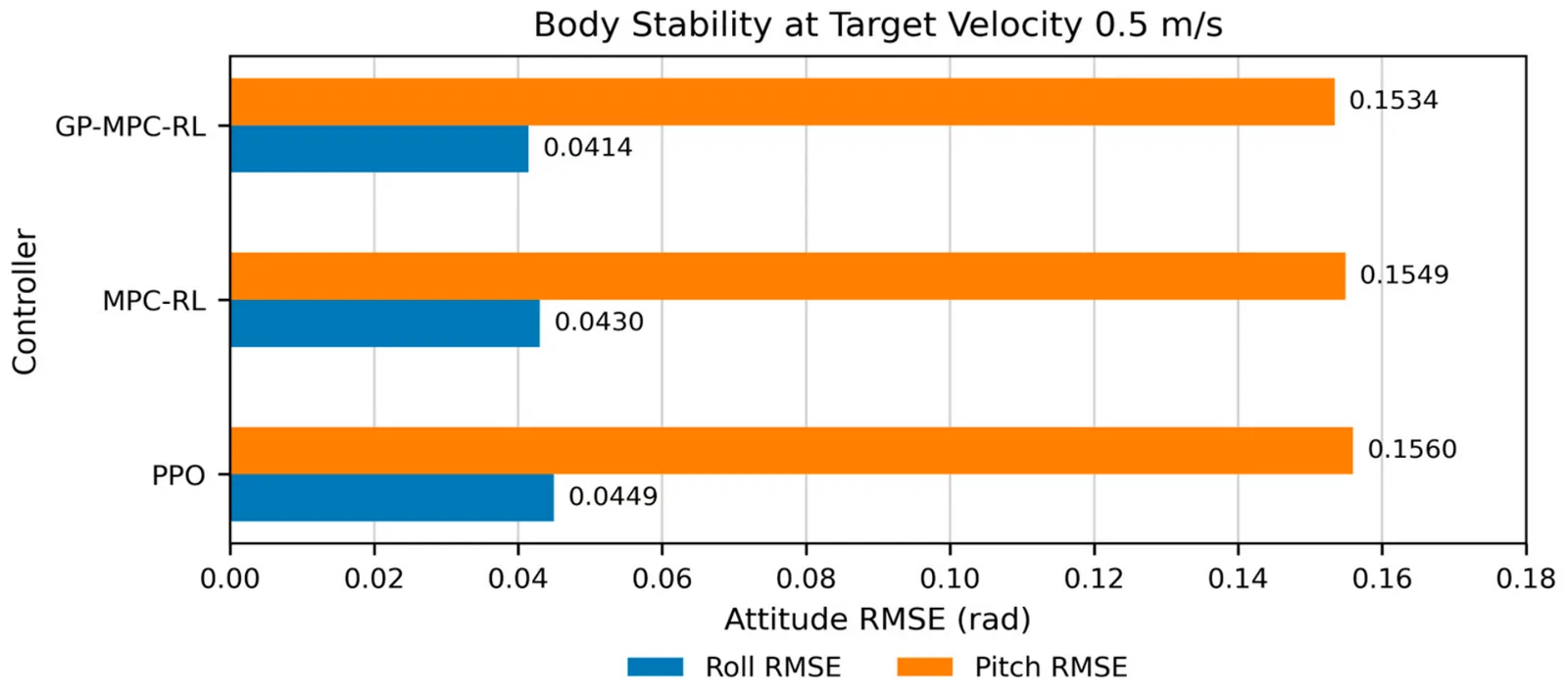
Roll and pitch RMSE at a target velocity of 0.5 m/s over 20 rough-terrain trials. Bars indicate the mean, and error bars indicate one standard deviation.

At 0.7 m/s, the roll RMSE values were 0.0435 ± 0.0194, 0.0385 ± 0.0140, and 0.0392 ± 0.0127 rad for PPO, MPC-RL, and GP-MPC-RL, respectively. The corresponding pitch RMSE values were 0.1742 ± 0.0896, 0.1759 ± 0.0908, and 0.1742 ± 0.0842 rad. Overall, GP-MPC-RL maintained body-attitude regulation comparable to MPC-RL across the tested walking speeds, while preserving the forward-velocity tracking improvements provided by command-level supervision. A representative comparison of roll and pitch RMSE at 0.5 m/s is shown in Figure 12.

Overall, the supervisory controllers improved forward-velocity tracking without producing a systematic degradation in body attitude. However, GP compensation did not uniformly improve roll and pitch errors under nominal walking conditions. This observation is consistent with the controller design, which primarily applies the learned residual correction to the longitudinal command-response dynamics.

### 5.4. Robustness to Payload-Induced Dynamics Variations

The target forward velocity was fixed at 0.5 m/s, and additional masses of 0, 2, and 5 kg were applied to the robot base without introducing an intentional center-of-mass offset. Both MPC-RL and GP-MPC-RL were evaluated using the same set of 20 random seeds under all payload conditions, enabling paired comparisons.

Table 14 summarizes the behavior of GP-MPC-RL as the payload increased. The forward-velocity RMSE increased from 0.0590 ± 0.0142 m/s at 0 kg to 0.0624 ± 0.0312 m/s at 2 kg and 0.0914 ± 0.0759 m/s at 5 kg. The mean absolute longitudinal residual also increased under the largest payload, from 0.01551 ± 0.00305 m/s at 0 kg to 0.01992 ± 0.01356 m/s at 5 kg. This increase indicates that the added mass introduced a larger mismatch between the nominal command-response prediction and the measured robot dynamics.

Table 15 directly compares MPC-RL and GP-MPC-RL. Under the nominal 0 kg condition, the two methods exhibited nearly identical velocity-tracking performance: 0.0599±0.0144 m/s for MPC-RL and 0.0590±0.0142 m/s for GP-MPC-RL. At 2 kg, the RMSE values were 0.0659±0.0474 and 0.0624±0.0312 m/s, respectively. These differences were small, indicating that GP compensation provides limited additional benefit when the nominal model mismatch remains modest.

A substantially larger difference emerged under the 5 kg payload. MPC-RL produced a forward-velocity RMSE of 0.1288±0.1239 m/s, whereas GP-MPC-RL reduced the error to 0.0914±0.0759 m/s, corresponding to a 29.0% reduction. GP-MPC-RL achieved a lower RMSE in 14 of the 20 paired trials. Because the paired differences were strongly non-normal (Shapiro–Wilk p<0.001), the Wilcoxon signed-rank test was used as the primary comparison and indicated a significant difference (W=45, p=0.024). The paired t-test showed the same directional trend but did not reach the conventional significance threshold ($t\left(19\right)=1.93$, p=0.069).

The longitudinal model residual exhibited a similar pattern. At 5 kg, the mean absolute residual decreased from 0.02692±0.02606 m/s for MPC-RL to 0.01992±0.01356 m/s for GP-MPC-RL, corresponding to an approximately 26.0% reduction. The roll RMSE was also reduced from 0.0874 ± 0.1112 rad to 0.0471 ± 0.0197 rad, while the pitch RMSE decreased from 0.1984 ± 0.0828 rad to 0.1787 ± 0.0504 rad.

**Figure 13:**
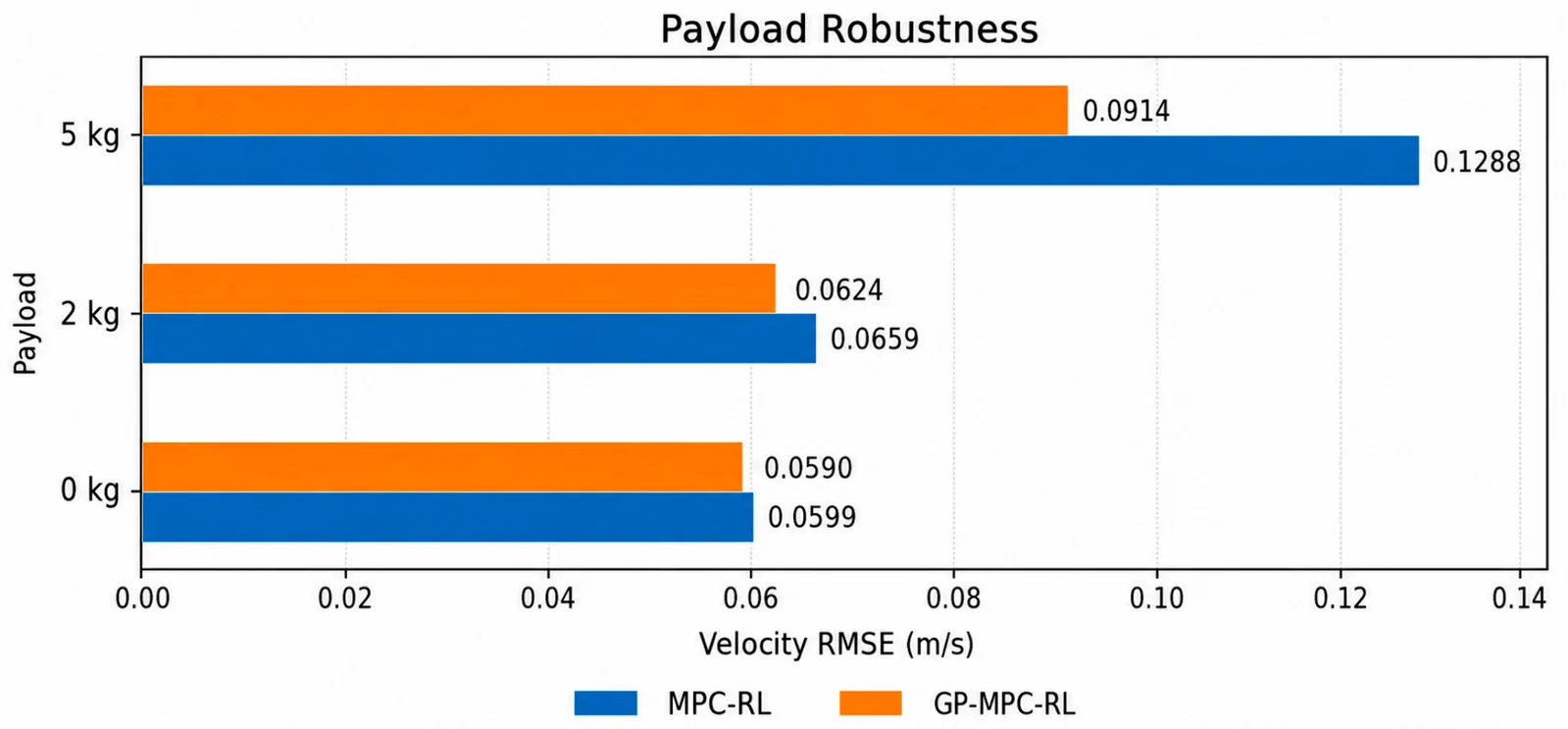
Forward-velocity RMSE of MPC-RL and GP-MPC-RL under 0, 2, and 5 kg payload conditions at a target velocity of 0.5 m/s. Each bar represents the mean over 20 trials.

To quantify robustness more directly, the payload-induced degradation was defined as the increase in velocity RMSE from the 0 kg condition to the 5 kg condition for each paired seed. The mean degradation was 0.0689 ± 0.1133 m/s for MPC-RL and 0.0324 ± 0.0639 m/s for GP-MPC-RL. Thus, GP-MPC-RL reduced the mean payload-induced degradation by 53.0%. The degradation was smaller for GP-MPC-RL in 15 of the 20 paired trials, and the Wilcoxon signed-rank test indicated a significant reduction (W=43,p=0.019). These results support the central role of the GP residual model: its main benefit is not a uniform improvement under nominal conditions but increased robustness when the actual command-response dynamics deviate substantially from the nominal predictive model. The payload-dependent forward-velocity RMSE comparison is shown in Figure 13.

### 5.5. Computational Performance

The average supervisory computation times measured over the 60 fixed-velocity trials (20 trials at each of 0.3, 0.5, and 0.7 m/s) are summarized in Table 16.

Across the three target-velocity conditions, MPC-RL required 0.0701 ± 0.0035 ms per supervisory update, whereas GP-MPC-RL required 0.1117 ± 0.0063 ms. The GP residual processing therefore introduced an average additional computation time of approximately 0.0416 ms, corresponding to a relative increase of approximately 59.3%. Although the relative increase was noticeable, the absolute additional cost remained below 0.05 ms per supervisory update.

The reported values represent only supervisor-level computation, including optimization and, for GP-MPC-RL, GP-related processing measured by the implemented logging framework. They do not represent the complete end-to-end latency of the control system, which additionally includes policy inference, sensor processing, physics simulation, communication, and low-level control.

### 5.6. Overall Discussion

The experimental results reveal three main characteristics of the proposed GP-MPC-RL framework.

First, command-level predictive supervision consistently improved forward-velocity tracking relative to the original PPO locomotion policy over the tested walking-speed range. At target velocities of 0.3, 0.5, and 0.7 m/s, GP-MPC-RL reduced the mean forward-velocity RMSE by approximately 38.3%, 21.3%, and 10.1%, respectively, relative to PPO. Compared with MPC-RL, the additional reductions provided by GP-MPC-RL were 10.4%, 1.5%, and 6.4%, respectively. The Wilcoxon signed-rank test indicated a significant additional improvement over MPC-RL at 0.3 m/s (p=0.017), whereas the differences at 0.5 and 0.7 m/s were not statistically significant. These results indicate that the nominal MPC supervisor accounts for a substantial portion of the tracking improvement under nominal operating conditions, while the GP residual term provides an additional but generally modest benefit when the nominal command-response model already represents the locomotion dynamics reasonably well.

Second, the benefit of GP residual compensation became substantially more pronounced when the nominal model was subjected to payload-induced dynamics mismatch. At 0 kg, MPC-RL and GP-MPC-RL produced nearly identical forward-velocity RMSE values of 0.0599±0.0144 and 0.0590±0.0142 m/s, respectively. A similarly small difference was observed at 2 kg. Under the 5 kg payload, however, the RMSE increased to 0.1288±0.1239 m/s for MPC-RL, whereas GP-MPC-RL limited the error to 0.0914±0.0759 m/s, corresponding to a 29.0% reduction. The mean absolute longitudinal residual was also reduced by approximately 26.0%, from 0.02692 to 0.01992 m/s. The Wilcoxon signed-rank test indicated a significant paired reduction in forward-velocity RMSE at 5 kg (W=45, p=0.024), although the corresponding paired t-test did not reach the conventional significance threshold (p=0.069) because the paired differences were strongly non-normal.

The robustness effect becomes clearer when the degradation caused by the added payload is considered directly. From 0 to 5 kg, the mean increase in velocity RMSE was 0.0689±0.1133 m/s for MPC-RL and 0.0324±0.0639 m/s for GP-MPC-RL. Thus, GP-MPC-RL reduced the mean payload-induced degradation by approximately 53.0%. The degradation was smaller for GP-MPC-RL in 15 of the 20 paired trials, and the Wilcoxon signed-rank test indicated a significant difference (W=43, p=0.019). This result supports the central hypothesis of the proposed method: the GP residual model is most beneficial when the measured robot response departs substantially from the nominal command-response prediction.

The payload experiments also showed that the improved forward-velocity tracking was not obtained at the expense of body-attitude regulation. Under the 5 kg condition, GP-MPC-RL reduced the mean roll RMSE from 0.0874±0.1112  to 0.0471±0.0197 rad and the mean pitch RMSE from 0.1984±0.0828 to 0.1787±0.0504 rad. Under nominal walking conditions, however, the roll and pitch differences among PPO, MPC-RL, and GP-MPC-RL were generally small and did not exhibit a uniform ranking. Therefore, the main contribution of the proposed framework should be interpreted as improved velocity-tracking robustness under dynamics mismatch rather than universal improvement in all body-attitude metrics.

Third, the GP-enhanced supervisor retained a low computational cost. Across the fixed-velocity experiments, the average supervisor-level computation time increased from approximately 0.0701±0.0035 ms for MPC-RL to 0.1117±0.0063 ms for GP-MPC-RL. Although this corresponds to a relative increase of approximately 59.3%, the absolute additional cost was only about 0.0416 ms per supervisory update. The reported timing represents supervisor-level optimization and GP processing rather than the total end-to-end latency of the complete locomotion system.

Overall, the results show that GP-MPC-RL does not simply provide a uniform improvement over nominal MPC-RL in every operating condition. Instead, its advantage becomes increasingly evident as the discrepancy between the nominal predictive model and the measured robot dynamics grows. This behavior is consistent with the intended role of the GP residual model as an online correction mechanism for model mismatch while preserving the pretrained PPO locomotion policy.

Several limitations remain. The current validation was conducted entirely in simulation, and the payload was applied as additional base mass without explicitly shifting the center of mass. The evaluation also focused primarily on forward-velocity tracking, and no formal closed-loop stability guarantee is provided for the combined learning-based supervisory framework. Future work will therefore include hardware experiments, asymmetric payloads, external disturbances, sensor noise, and explicit evaluation of end-to-end timing and contact uncertainty. The present command-level supervisor does not explicitly model rapid limb-level reflexes or contact-force-based balance recovery; such local reactive mechanisms could complement the proposed predictive supervisory layer, particularly under abrupt foothold loss or slipping.

## 6. Conclusions

This study proposed a GP-MPC-RL supervisory control framework for reinforcement learning-based quadruped locomotion on uneven terrain. The framework combines a pretrained and frozen PPO locomotion policy with an acados-based command-level MPC supervisor and a GP residual model. The GP estimates the discrepancy between the nominal command-response prediction and the measured robot response, and the learned residual correction is incorporated into the supervisory prediction without modifying the underlying locomotion policy.

The proposed framework was evaluated in Isaac Lab using a Unitree Go2 quadruped model. Forward-velocity tracking was tested at target velocities of 0.3, 0.5, and 0.7 m/s using 20 paired trials for each operating condition. GP-MPC-RL achieved mean forward-velocity RMSE values of 0.0411±0.0109, 0.0590±0.0142, and 0.0883±0.0349 m/s, respectively. Relative to PPO, these values correspond to reductions of approximately 38.3%, 21.3%, and 10.1%. Relative to MPC-RL, the additional reductions were 10.4%, 1.5%, and 6.4%. These results show that command-level MPC supervision provides a clear improvement over the original PPO policy, whereas the additional contribution of the GP residual model under nominal operating conditions is comparatively modest.

The importance of the GP residual correction became more evident under payload-induced dynamics mismatch. At a target velocity of 0.5 m/s and a 5 kg payload, GP-MPC-RL reduced the mean forward-velocity RMSE from 0.1288±0.1239 m/s for MPC-RL to 0.0914±0.0759 m/s, corresponding to a 29.0% reduction. The mean absolute longitudinal residual was reduced by approximately 26.0%, and the roll and pitch RMSE were reduced by approximately 46.1% and 9.9%, respectively.

More importantly, the increase in forward-velocity RMSE from 0 to 5 kg was reduced from 0.0689±0.1133 m/s for MPC-RL to 0.0324±0.0639 m/s for GP-MPC-RL. This represents a 53.0% reduction in mean payload-induced degradation. GP-MPC-RL exhibited smaller degradation in 15 of the 20 paired trials, with a statistically significant Wilcoxon signed-rank result (W=43, p=0.019). These findings indicate that the principal benefit of the GP residual model arises when the actual locomotion response departs substantially from the nominal predictive model.

The additional computational requirement of the GP-enhanced supervisor remained small in absolute terms. The mean supervisor-level computation time increased from approximately 0.0701 ms for MPC-RL to 0.1117 ms for GP-MPC-RL, corresponding to an additional cost of approximately 0.0416 ms per supervisory update. These values indicate that the GP correction can be incorporated into the command-level optimization with limited additional computational burden in the simulation environment.

The present study is limited to simulation-based validation. In addition, the payload was modeled as an added base mass without explicitly shifting the center of mass, and the current controller primarily targets longitudinal command-response mismatch. No formal stability guarantee is claimed for the combined GP-MPC-RL framework. Future work will focus on hardware validation using a physical quadruped robot, evaluation under asymmetric payloads and external disturbances, incorporation of angular and contact-related residual models, and end-to-end timing analysis on onboard computing hardware.

Overall, the results demonstrate that GP-MPC-RL is most effective as a model-mismatch compensation mechanism rather than as a uniformly superior replacement for nominal MPC supervision. By retaining the pretrained RL locomotion policy while adapting the predictive supervisory model to observed dynamics errors, the proposed framework improves locomotion robustness under substantial unmodeled dynamics without requiring retraining of the underlying policy.

## Figures and Tables

**Table 1:** Simulation and control settings.

Parameter	Value
Simulator	Isaac Sim 5.1
Physics Engine	NVIDIA PhysX
Robot Platform	Unitree Go2
Number of degrees of freedom (DOFs)	12
Simulation Frequency	200 Hz
Low-Level Control Frequency	50 Hz
Command-supervisor frequency	25 Hz
Supervisor interval	0.04 s
Terrain Types	Flat, Gravel, Slope, Step

**Table 2:** PPO training parameters.

Parameter	Value
RL Algorithm	PPO
Discount Factor γ	0.99
GAE λ	0.95
Learning Rate	3 × 10^−4^
PPO Clip Ratio	0.2
Entropy Coefficient	0.01
Value Loss Coefficient	0.5
Mini-batch Size	4096
Epochs	5
Total Timesteps	5 × 10^7^
Forward-velocity command range	0.0~0.8 m/s
Yaw-rate command range	−0.8~0.8 rad/s

**Table 3:** GP hyperparameters and residual-correction settings used in the experiments.

Parameter	Value
GP input dimension	19
GP output dimension	6
Kernel	RBF/squared exponential
Length scale l	0.8
$\mathrm{Signal}\;\mathrm{variance}\;\sigma_{f}^{2}$	1.0
$\mathrm{Noise}\;\mathrm{variance}\;\sigma_{n}^{2}$	$1.0\times 10^{-3}$
Residual clipping	[−0.5, 0.5], element-wise
GP correction gain	1.5
Correction clipping	[−0.15, 0.15]
Confidence factor	$c=exp(-2\bar{\sigma_{GP}})$
Residual applied to MPC	$\mathrm{Longitudinal}\;\mathrm{velocity}\;\mathrm{residual}\;r_{vx}$ only
Minimum samples before GP prediction	12
Maximum retained GP samples	200
GP refit interval	5 updates
Horizon residual-decay factor	0.80

**Table 4:** Compared control methods.

Method	PPO Policy	GP Residual	MPC Supervisor	Description
RL-only	✓	✗	✗	Frozen PPO policy without command supervision
Nominal MPC + RL	✓	✗	✓	PPO supervised using the nominal command-response model
Proposed GP-MPC + RL	✓	✓	✓	PPO supervised using the proposed GP-corrected prediction model

Note: ✓ indicates that the component is used; ✗ indicates that it is not used.

**Table 5:** Representative terrain environments considered in the simulation study.

Scenario	Terrain	Target Velocity
S1	Flat Ground	0.3, 0.5, 0.7 m/s
S2	Random uneven terrain, h∈[−0.05,0.05] m, grid resolution 0.10 m	0.3, 0.5, 0.7 m/s
S3	15° Uphill Slope	0.3, 0.5, 0.7 m/s
S4	10 cm Step Obstacle	0.3, 0.5, 0.7 m/s

**Table 6:** Geometric properties of the generated terrains.

Terrain	Height std. (m)	Slope std. (deg)	Maximum Slope (deg)	Mean Local Roughness (m)
Mild	0.010	0.353	2.089	0.000447
Medium	0.025	0.883	5.210	0.001118
Severe	0.050	1.759	10.336	0.002236

Note: std., standard deviation.

**Table 7:** Statistical properties of the transition residuals.

Residual Component	Mean	Standard Deviation
$\Delta v_{x}\;(m/s)$	$-1.103\times 10^{-6}$	$5.385\times 10^{-5}$
$\Delta v_{y}\;(m/s)$	$-2.360\times 10^{-7}$	$5.345\times 10^{-5}$
$\Delta v_{z}\;(m/s)$	$9.079\times 10^{-2}$	$7.048\times 10^{-2}$
$\Delta \omega_{x}\;(rad/s)$	$-1.956\times 10^{-2}$	$4.163\times 10^{-1}$
$\Delta \omega_{y}\;(rad/s)$	$-2.834\times 10^{-2}$	$4.122\times 10^{-1}$
$\Delta \omega_{z}\;(rad/s)$	$-5.599\times 10^{-6}$	$1.231\times 10^{-3}$

**Table 8:** Predictive-controller and solver configuration.

Parameter	Value
Prediction model	12-state rigid-body model
State dimension	12
Input dimension	6
Prediction horizon	10
Sampling interval	0.05 s
Prediction duration	0.5 s
NLP method	SQP_RTI
QP solver	Partial-condensing HPIPM
Symbolic Modeling	CasADi
Solver implementation	acados (version 0.6.0)

**Table 9:** Controller configurations used in the terrain-conditioned validation.

Method	Nominal Prediction	GP Residual	Continuous Terrain Descriptor
MPC	✓	✗	✗
GP-MPC	✓	✓	✗
Terrain-conditioned GP-MPC	✓	✓	✓

Note: ✓ indicates that the component is used; ✗ indicates that it is not used.

**Table 10:** Performance of the compared controllers under mild, medium, and severe terrain conditions.

Terrain	Method	Velocity RMSE	Roll RMSE	Pitch RMSE	Smoothness	Solve Time (ms)	Tracking Tolerance Rate
Mild	MPC	0.0372	0.0144	0.0309	0.0871	0.0584	1.000
	GP-MPC	0.0329	0.0043	0.0093	0.0211	0.00549	1.000
	Terrain-conditioned GP-MPC	0.0326	0.0247	0.0577	0.0111	0.0812	1.000
Medium	MPC	0.0613	0.0359	0.07772	0.2584	0.0592	0.894
	GP-MPC	0.0340	0.0108	0.0232	0.0469	0.0573	1.000
	Terrain-conditioned GP-MPC	0.0326	0.0257	0.0567	0.0110	0.0832	1.000
Severe	MPC	0.1006	0.0718	0.1544	0.7449	0.0762	0.678
	GP-MPC	0.0364	0.0215	0.00463	0.0802	0.0573	1.000
	Terrain-conditioned GP-MPC	0.0327	0.0274	0.0549	0.0110	0.0775	1.000

**Table 11:** Forward-velocity tracking RMSE on fixed rough terrain.

Controller	0.3 m/s	0.5 m/s	0.7 m/s
PPO	0.0666 ± 0.0248	0.0750 ± 0.0204	0.0982 ± 0.0424
MPC_RL	0.0459 ± 0.0215	0.0599 ± 0.0144	0.0943 ± 0.0411
GP-MPC-RL	0.0411 ± 0.0109	0.0590 ± 0.0142	0.0883 ± 0.0349

**Table 12:** Supervisory command correction, mean absolute longitudinal residual, and supervisor-level computation time at different target velocities. Values are reported as mean ± SD over 20 trials.

Controller	Target Velocity	Command RMSE (m/s)	Mean ∣*r_vx_*∣ (m/s)	Supervisory Computation Time (ms)
PPO	0.3 m/s	0.0000 ± 0.0000	0.01292 ± 0.00394	N/A
	0.5 m/s	0.0000 ± 0.0000	0.01494 ± 0.00372	N/A
	0.7 m/s	0.0000 ± 0.0000	0.01784 ± 0.00531	N/A
MPC-RL	0.3 m/s	0.03968 ± 0.01020	0.01288 ± 0.00315	0.0681 ± 0.0016
	0.5 m/s	0.04508 ± 0.01287	0.01541 ± 0.00340	0.0688 ± 0.0018
	0.7 m/s	0.04024 ± 0.01165	0.01873 ± 0.00588	0.0735 ± 0.0036
GP-MPC-RL	0.3 m/s	0.04481 ± 0.01018	0.01274 ± 0.00251	0.1079 ± 0.0015
	0.5 m/s	0.04875 ± 0.01224	0.01551 ± 0.00305	0.1087 ± 0.0023
	0.7 m/s	0.04252 ± 0.01262	0.01846 ± 0.00522	0.1186 ± 0.0066

Note: N/A, not applicable.

**Table 13:** Body-attitude RMSE at different target velocities.

Controller	Target Velocity	Roll RMSE (rad)	Pitch RMSE (rad)
PPO	0.3 m/s	0.0559 ± 0.0117	0.1414 ± 0.0696
MPC-RL	0.3 m/s	0.0499 ± 0.0085	0.1427 ± 0.0530
GP-MPC-RL	0.3 m/s	0.0512 ± 0.0084	0.1496 ± 0.0631
PPO	0.5 m/s	0.0449 ± 0.0136	0.1560 ± 0.0763
MPC-RL	0.5 m/s	0.0430 ± 0.0112	0.1549 ± 0.0729
GP-MPC-RL	0.5 m/s	0.0414 ± 0.0091	0.1534 ± 0.0696
PPO	0.7 m/s	0.0435 ± 0.0194	0.1742 ± 0.0896
MPC-RL	0.7 m/s	0.0385 ± 0.0140	0.1759 ± 0.0908
GP-MPC-RL	0.7 m/s	0.0392 ± 0.0127	0.1742 ± 0.0842

**Table 14:** GP-MPC-RL performance under increasing payload.

Payload	Velocity RMSE	Roll RMSE	Pitch RMSE	Mean ∣*r_vx_*∣ (m/s)	Supervisory Computation Time (ms)
0 kg	0.0590 ± 0.0142	0.0414 ± 0.0091	0.1534 ± 0.0696	0.01551 ± 0.00305	0.1087 ± 0.0023
2 kg	0.0624 ± 0.0312	0.0454 ± 0.0107	0.1583 ± 0.0685	0.01397 ± 0.00457	0.1087 ± 0.0023
5 kg	0.0914 ± 0.0759	0.0471 ± 0.0197	0.1787 ± 0.0504	0.01992 ± 0.01356	0.1118 ± 0.0071

**Table 15:** Comparison of MPC-RL and GP-MPC-RL under 0, 2, and 5 kg payload conditions at a target forward velocity of 0.5 m/s. Values are reported as mean ± SD over 20 paired trials.

Controller	Payload (kg)	Velocity RMSE (m/s)	Roll RMSE (rad)	Pitch RMSE (rad)	Mean ∣*r_vx_*∣ (m/s)	Supervisory Computation Time (ms)
MPC-RL	0	0.0599 ± 0.0144	0.0430 ± 0.0112	0.1549 ± 0.0729	0.01541 ± 0.00340	0.0688 ± 0.0018
GP-MPC-RL	0	0.0590 ± 0.0142	0.0414 ± 0.0091	0.1534 ± 0.0696	0.01551 ± 0.00305	0.1087 ± 0.0023
MPC-RL	2	0.0659 ± 0.0474	0.0467 ± 0.0121	0.1656 ± 0.0814	0.01488 ± 0.00861	0.0685 ± 0.0017
GP-MPC-RL	2	0.0624 ± 0.0312	0.0454 ± 0.0107	0.1583 ± 0.0685	0.01397 ± 0.00457	0.1087 ± 0.0023
MPC-RL	5	0.1288 ± 0.1239	0.0874 ± 0.1112	0.1984 ± 0.0828	0.02692 ± 0.02606	0.0733 ± 0.0062
GP-MPC-RL	5	0.0914 ± 0.0759	0.0471 ± 0.0197	0.1787 ± 0.0504	0.01992 ± 0.01356	0.1118 ± 0.0071

**Table 16:** Average supervisory computation time.

Controller	Supervisory Computation Time (ms)
MPC-RL	0.0701±0.0035
GP-MPC-RL	0.1117±0.0063

## Data Availability

The datasets generated and analyzed during the current study are available from the corresponding author upon reasonable request.

## Abbreviations

GP	Gaussian Process
MPC	Model Predictive Control
NMPC	Nonlinear Model Predictive Control
PPO	Proximal Policy Optimization
RL	Reinforcement Learning
RMSE	Root Mean Square Error
IK	Inverse Kinematics
PD	Proportional–derivative
SRB	Single Rigid Body
COM	Center of Mass
DoF	Degree of Freedom
